# From violation to stigma: a literature review of athletes' lived experiences following anti-doping sanctions

**DOI:** 10.3389/fspor.2026.1651135

**Published:** 2026-02-26

**Authors:** Isaac Lockett, Cornelia Blank, Laurie Patterson, Daniel Westmattelmann, Daniela Lux, Andrea Petróczi

**Affiliations:** 1 School of Life Sciences, Pharmacy and Chemistry, Faculty of Health, Science, Social Care and Education, Kingston University, Kingston upon Thames, United Kingdom; 2 Department of Sport Science, University of Innsbruck, Innsbruck, Austria; 3 Carnegie School of Sport, Leeds Beckett University, Leeds, United Kingdom; 4 Center for Management, University of Münster, Münster, Germany; 5 Private University of Applied Sciences for Business and Technology, Vechta, Germany; 6 Institute of Health Promotion and Sport Sciences, Faculty of Education and Psychology, ELTE Eötvös Loránd University, Budapest, Hungary

**Keywords:** anti-doping, athlete, ineligibility, justice, legitimacy, rule violation, sanction, sport

## Abstract

Despite its protective intent, the anti-doping system functions in a complex, high-stakes environment where strict liability and public scrutiny can produce unintended harms for sanctioned athletes. To date, limited insights exist into the lived experiences of individuals sanctioned for Anti-Doping Rule Violations (ADRVs) under the World Anti-Doping Code. We located twelve English-language outputs published between 2011 and 2025 containing relevant empirical data, and used descriptive and bibliometric techniques, narrative synthesis, and citational analysis to evaluate the composition, impact, and practical relevance of this emerging body of work. Through narrative synthesis, we identified five key areas of concern: (1) psychological and emotional distress; (2) social isolation and reputational damage; (3) perceived procedural injustice and institutional abandonment; (4) disruption to career and athletic identity; and (5) a sense of resolution or clarity despite adversity. Difficulty in reaching and recruiting participants was noted in most studies. Highly cited articles were referenced across diverse domains, often due to their dual focus on doping motivations and firsthand experiences with ADRV sanctions. The direct translation of existing research into anti-doping policy remains limited. Gaps in reporting standards, the underrepresentation of female athletes, and regional imbalances in the evidence base constrain a comprehensive understanding of the personal consequences of ADRV sanctions. Despite these early limitations, this review marks a critical step toward evaluating the legitimacy of anti-doping rule enforcement from the perspective of those most directly affected. The findings highlight the urgent need for future research to inform more humane, equitable, and athlete-centred regulatory practices.

## 1 Introduction

The World Anti-Doping Agency (WADA) is a legitimacy-based authority (1, 2), with the authority of the organization dependent on the continued recognition of a rightful mandate to regulate sports-related doping (3). Structurally, WADA operates as a top-down organization (4), positioning itself as the primary policy maker and overseer within the World Anti-Doping Program” (5, p. 9). The organization operationalizes this structure through the World Anti-Doping Code (WADC), which establishes the rules, standards, and processes that signatory organizations, such as National Anti-Doping Organizations (NADO) and international federations, are required to implement. These signatory organizations, which operate across diverse socio-geographic, political, organizational, and resource environments (1), inherit responsibility for the day-to-day operation of the World Anti-Doping Program under the oversight of WADA. Signatories that fail to uphold this responsibility risk sanctioning, reinforcing WADA's position as policy overseer. This structure creates an environment whereby assessing WADA as a regulatory authority requires an understanding of how the WADA maintained macro-level policy environment and the micro-level implementation practices of signatory organizations, for which WADA retains de facto responsibility, are perceived by those governed (6).

Understanding how athletes governed by the WADC perceive both environments is of particular importance for WADA (2). Failure to maintain athletes’ perceptions of the World Anti-Doping Program risks increasing resistance, non-compliance, and erosion of normative authority (7). The three dimensions of perceived legitimacy (2, 7) provide a meaningful framework for evaluating these perspectives: normative legitimacy (the governed population accepts that governance is needed and the authority has the right to govern), performance legitimacy (the governed population agrees that the authority's rules achieve the intended outcomes), and procedural legitimacy (the governed population feels that the authority applies its rules fairly, consistently, and produces fair outcomes). When assessing a legitimacy-based authority, it is also important to consider how the authority anticipates and mitigates unintended harms for those subject to its governance (8). This includes evaluating the proportionality of sanctioning (9, 10) and assessing whether adequate safeguards are in place and functional (11). For WADA specifically, it is also important to consider the human costs involved in maintaining clean sport (12, 13).

Further nuance is required when assessing the perceived legitimacy of WADA as an authority and the WADC as a regulatory framework, given that athletes’ exposure to specific WADC Articles varies. The WADC is a collection of interrelated Articles that, while collectively contributing to the World Anti-Doping Program, function as independent policies and practices. It is therefore necessary to consider how athletes who have been exposed to specific Articles of the WADC describe their lived experiences of the related processes and outcomes, as these perspectives provide insight into how WADA's rules and the related implementation practices are experienced in everyday practice, rather than relying on assumptions or the views of individuals without direct lived experience (14). For example, not all athletes are exposed to the sanctioning process outlined in the WADC. Articles 2, 7, 10, 11 provide the core framework (5). Article 2 defines the list of Anti-Doping Rule Violations; Article 7 outlines the results management process, including procedural steps following an Adverse Analytical Finding, the notification process and provisional suspension provisions; Articles 10 outlines the sanctions applicable to individuals, with potential sanctions including the disqualification of results, periods of ineligibility, the forfeiture of prize money, the inability to access training facilities or to return to team environments until the reintegration window at the end of the sanction. This sanctioning framework, in particular Article 10, represents the most severe intervention in the WADC, making it both a crucial focus for assessing legitimacy and an area that requires specific investigation, as the resulting impacts can only be understood through the experiences of athletes who have been subject to it.

Despite this importance, the perspectives of athletes who have been exposed to the WADC sanctioning process remain under-represented in research. Currently, insights on ADRV sanctioning have been generated from theoretical positions or from athletes whose exposure to the WADC sanctioning framework is unclear, which, while providing important conceptual and contextual understanding, does not integrate the lived experiences of athletes or offer insight into how the sanctioning framework is experienced in practice, and thus does not illuminate athletes’ perceptions of the legitimacy of the process [e.g., (15–22)]. Alongside this literature, a limited number of published materials have captured the perspectives of athletes directly affected by the WADC sanctioning framework, providing valuable insight [e.g., (23–25)]. These insights remain fragmented and have had limited influence on policymaking (26). A systematic consolidation of this evidence may strengthen its potential impact by providing policymakers with a comprehensive, rather than fragmented, understanding of how sanctions are experienced. This review therefore focuses on consolidating insights from athletes with lived experience of being sanctioned for an ADRV under the WADC, aiming to generate a coherent evidence base to examine the consequences of sanctioning and support ongoing policy discussions.

## 2 Aims

This review critically examines and synthesizes existing evidence that provides insight into the lived experiences of athletes who have been exposed to the WADC ADRV sanctioning process. Specifically, it seeks to address the following research questions:

How do athletes describe their experiences of being sanctioned under the WADA-established ADRV sanctioning process?
How do athletes perceive the WADC sanctioning process and its outcomes in terms of procedural fairness, outcome fairness, and broader life impacts, and how have these experiences been interpreted by researchers in relation to legitimacy?
What patterns and gaps exist in the published research examining the experiences of athletes exposed to the WADC sanctioning process?
How has research into the experiences of athletes exposed to the WADC sanctioning process influenced developments in anti-doping research and policy?

## 3 Methods

To address the research questions, an inclusive and exploratory approach to reviewing literature was adopted. Rather than limiting the review to empirical studies, this approach enabled the inclusion of a broader range of outputs while maintaining a focus on athletes who have experienced the WADC sanctioning process. Identified outputs were systematically analyzed using bibliometric and narrative methods, enabling assessment of their content, representational depth, and impact.

### 3.1 Research context

This review forms part of the TALE and What About Us? projects (https://www.athletes-tale.eu), which were developed in response to the recognition that while considerable attention is devoted to detecting and sanctioning doping, comparatively little is directed toward the human cost of these actions. The projects were conceptualized following the introduction of WADA's International Standard for Education, which states that evidence-informed education should be provided to athletes returning from an ADRV sanction (5). Aiming to contribute to these discussions, the projects seek to identify research gaps and generate evidence to inform policy recommendations and practical guidelines that better prepare emerging athletes and support those facing sanctions.

### 3.2 The research team

The authors of this review bring a diverse and complementary range of expertise spanning psychology, sport psychology, sport science, public health, sports business management, and social science–focused anti-doping research. The first author, with a background in sport psychology and sports business management, brings experience in document analysis and evidence synthesis. The second, third, fourth and sixth authors are established experts in social science–focused anti-doping research, recognized internationally for their contributions to the field. The second, third, fifth, and sixth authors have contributed to research examining the lived experience of athletes sanctioned under the WADC, including outputs reviewed as part of this study. These collective perspectives are strengthened by the fourth author, a former professional athlete and anti-doping researcher with a background in sports management and social science.

Prioritizing the practical consequences and the real-world implications of anti-doping sanctioning, our work is grounded in a pragmatist research philosophy (27). As a multidisciplinary team, the authorship group acknowledges that our interpretations of the reviewed outputs are shaped by both proximity to and critical engagement with the anti-doping system. These perspectives provide valuable contextual sensitivity but also position us within a field where regulatory narratives, athlete welfare, and institutional credibility coexist in tension. Adopting a pragmatist stance allows us to balance these influences by focusing on the lived consequences of ADRVs to inform and improve regulatory practices. Our aim is neither to defend nor to discredit the anti-doping system, but to generate actionable insights that support fair processes to all involved and reduce unintended harms. This orientation recognizes the complex, high-stakes environment in which anti-doping operates and foregrounds the experiences of those most affected, while remaining attentive to gaps, uncertainties, and the limitations inherent in the available evidence.

### 3.3 Output selection

Initially, a keyword-based search strategy was developed to identify relevant outputs. However, this strategy proved unsuitable as despite multiple revisions, searches consistently generated large numbers of irrelevant results and failed to retrieve outputs previously identified by the research team (Supplementary Table 1.1). In response, an iterative multi-phase search strategy was developed and adopted, consistent with exploratory literature review style inquires (28–30).

The output identification process involved:

Existing knowledge: Cataloguing relevant literature known to the authorship group;
Iterative identification: Actively monitoring and collecting relevant published sources from 2022.
Snowballing: Reviewing reference lists (backward) and citations (forward).

Outputs identified to have potential relevance were compiled and compared against the inclusion and exclusion criteria by the first author. These criteria were developed according to the principles of sensitivity, which aimed to broadly capture potentially relevant outputs, and specificity, which ensured that included outputs aligned with the research objectives. In cases where inclusion could not be determined, outputs were referred to the last author, who made the final determination. The list of included outputs was finalized on 2 December 2025.

#### 3.3.1 Inclusion criteria

Outputs were eligible for inclusion if the following conditions were met:

Type of publication: Peer-reviewed research articles, peer-reviewed book chapters, pre-prints, doctoral or master's theses, or publicly available research reports.
Focus: Reporting first-hand data on athletes’ experiences and perspectives following direct exposure to the WADC sanctioning framework.
Timeframe: Published after the introduction of the WADC in 2002.
Language: Published in English or accompanied by a published English translation.

#### 3.3.2 Exclusion criteria

Outputs were excluded if any of the following conditions were met:

Analytical focus: Presents statistical analyses of ADRVs or patterns in case decisions without addressing experiential aspects.
Behavioral focus: Concentrates exclusively on factors contributing to doping use, without exploring sanction-related experiences.
Support provision: Examines support for sanctioned athletes without incorporating the lived experience of athletes.
Legal or ethical focus: Examines legal or ethical aspects of doping sanctions without including the lived experiences of athletes.
Duplicate data: Presents data already published in other works.

### 3.5 Quality assessment

Following the completion of the output selection process, a quality assessment was conducted on the included outputs. This phase did not affect inclusion decisions and was not used to judge the inherent value of the outputs. Rather, the assessment examined adherence to best-practice standards for reporting qualitative data. The tool presented by Lockwood et al. (31) was adopted to complete this assessment because it was specifically designed for this purpose. Five authors (first, second, fourth, fifth, and sixth) independently scored eleven outputs using the tool, with responses coded as yes (1), no (0), unclear (0.5), or not applicable (missing). Given the format of Jamieson and Ordway (32), a case study presented in a book chapter, this output was excluded from the quality assessment (Supplementary Table 2.1).

Independent ratings were compiled, and interrater reliability was assessed by the second author using Fleiss’ kappa (33) and weighted Cohen's kappa for pairwise agreement (34). Discrepancies in ratings across outputs were discussed among the five authors to allow alternative interpretations of the appraisal criteria to be considered (Supplementary Table 2.2). The third author, an expert in qualitative methodologies, reviewed the four outputs with the highest inconsistency in the rating to provide a benchmark during these discussions. Consensus was not enforced, and differing views were retained where appropriate. Following this process, interrater reliability was reassessed and reported (Supplementary Table 2.3).

### 3.6 Charting, analyzing and reporting results

Outputs were coded both quantitatively and qualitatively, structured into three methodological strands:

Descriptive bibliometric analysis (Research Questions 3 and 4): Participant characteristics, temporal patterns, geographic coverage, and the disciplinary backgrounds of contributing authors were extracted and analyzed. Collaboration patterns among researchers producing the included outputs were also examined.

**Table 1:** Data extraction summary from the included outputs.

Study	Sample size	Sport(s)	Competitive level	Country	Time period	Sanction status/exposure	Method	Key findings
Engelberg et al. (35)	*n* = 2 athletes “detected” (within wider sample of 18 admitting doping)	Sport not specified for sanctioned athletes (wider sample included bodybuilding, powerlifting, cricket, basketball, rugby league, sprint kayak, swimming)	Not reported	Australia	Not reported	Detected via testing; sanction outcome not confirmed	Individual interviews	Limited guilt or stigma where doping perceived as normalised; expectation of detection; minimal judgement from immediate sporting environment
Erickson (36)	*n* = 1 (male)	American football	Student-athlete (university)	Not reported	Not reported	Serving a sanction relating to an adverse analytical finding (second year at interview)	Case study	Public nature of sanctions; professional and educational consequences; lack of institutional support; voicelessness
Georgiadis & Papazoglou (24)	*n* = 5 (3 male, 2 female; aged 22–29)	Individual sports (unspecified)	International/elite	Not reported	Interviews conducted 8–10 months post-ban	Sanctioned following failed doping controls (methyltrienolone or testosterone); all denied intentional use	Individual interviews	Shock and devastation; paranoia and blame; loss of trust in the system; feelings of injustice and bitterness; social, financial, and identity-related impacts
Hall et al. (25)	*n* = 2 (gender not reported)	Rugby union or rugby league	Professional	United Kingdom	Not reported	Sanctioned for substance-related ADRVs	Individual interviews	Loneliness and isolation; mental health impacts; importance of social networks; relief following sanction
Henning & Dimeo (37)	*n* = 1 athlete voice (from 66 sanctioned cases)	Cycling	Masters	United States	Not reported	Indirect exposure through unsuccessful TUE application; not formally sanctioned	Case study within a wider dataset	Emotional impact of TUE refusal; perceived unfairness; disparity in treatment; feeling unheard
Huseynli et al. (38)	*n* = 7 (5 male, 2 female)	Sport not specified	National and international	Not reported	Proceedings within previous 5 years	Athletes involved in anti-doping proceedings (pre-hearing, hearing, appeal) or serving sanctions	Individual interviews	Highly emotional processes; mixed perceptions of fairness; unequal treatment; voicelessness; both positive and negative experiences of hearings
Jamieson & Ordway (32)	*n* = 1 (male)	Surfing	Masters	New Zealand	2014–2015	Sanctioned for use/attempted use and possession (clenbuterol, dianabol); two-year sanction backdated by one year	Case study (CAS decision)	Dispute of sanction; lack of awareness of WADC obligations; no intent to enhance performance; absence of anti-doping education
Juma & Woolf (39)	*n* = 10 (6 male, 4 female)	Athletics (sprints, middle- and long-distance, hurdles, jumps)	Not reported	Kenya	Sanction dates not specified	All sanctioned for substance-related ADRVs (anabolic agents, beta-2 agonists, glucocorticoids, hormone and metabolic modulators)	Individual interviews	Differential impacts by socio-economic status; financial hardship; psychological distress including suicidal ideation; social consequences; personal development; lack of support
Kirby et al. (40)	*n* = 3 (all male; aged 29–46)	Sport not specified for sanctioned athletes (wider sample included cycling and weightlifting)	Professional or Olympic	Not reported	Not reported	One lifetime ban (second offence); two serving two-year sanctions	Individual interviews	Ridicule and loss of respect; breakdown of friendships; constrained employment prospects; mixed peer support
Piffaretti (23)	*n* = 11 (all male; mean age 27.6 ± 3.95)	Soccer, American football, basketball, bobsleigh, cycling, taekwondo, artistic gymnastics	Mixed competitive levels	European sample (Italy, German- and French-speaking Switzerland, Spain)	Not reported	Sanctioned for recreational and performance-enhancing substances; varied sanction durations	Individual interviews	Frustration with sanctioning processes; perceived unfairness; emotional distress; career disruption; loss of social networks; importance of family support; relief and fresh starts post-sanction
Shelley (41)	*n* = 2 (both male)	Distance running	Former international/professional	North Africa; North America	Not reported	Sanctioned for EPO; one serving four-year sanction, one completed 18-month sanction	Individual interviews	Negative social and mental health impacts; influence of social media on experiences
van der Kallen et al. (42)	*n* = 4 (gender not reported)	Sport not specified	National/international	Austria	2000–2019	Sanctioned for intentional ADRVs; all confessed	Individual interviews	Financial and professional consequences; relief at leaving the sporting system; negative mental and psychological impacts; confusion regarding state involvement and standard sanctions

Narrative synthesis (Research Questions 1 and 2): The lived experiences of athletes exposed to the WADC sanctioning framework were synthesized using a thematic summary approach (43). This synthesis drew on direct quotes from athletes as reported in the included outputs and the interpretations of those experiences provided by the original authors. Only data attributable to athletes with direct experience of the WADC sanctioning framework were extracted from outputs that included multiple participant groups to maintain analytic focus. No attempt was made to code or differentiate experiential data according to violation characteristics or athlete characteristics, including intentionality, as such distinctions were not consistently reported across included outputs and could not be reliably linked to individual experiential accounts. These accounts, both direct and interpreted, were then organized into recurring themes, points of divergence, and broader conceptual paradigms. Variation in context and richness of findings were retained rather than collapsed, supporting a nuanced account of athlete experiences. The coding and thematic development were initially conducted by the first author before being presented to and discussed with the other authors, ensuring transparency, consistency, and collective agreement in the synthesis.

Impact and connectivity analysis (Research Question 4): Within the context of this study, impact was defined as the combined academic visibility, disciplinary engagement, policy uptake, and conceptual emphasis of the field, measured through citation metrics and networks, policy citations and altmetrics, and keyword mapping. This definition was influenced by previous examinations of impact determinations (44, 45).

Academic impact was appraised using citation analysis, citation network analysis, and keyword mapping. Citation data were retrieved from Dimensions.AI, Scopus, and Clarivate (Web of Science). Citation frequency of each included output was analyzed quantitatively using raw citation counts, field-weighted citation impact (FWCI), and field citation ratios (FCR), which normalize citation counts relative to disciplinary and temporal baselines. Qualitative analysis examined the disciplinary orientation and thematic content of citing articles. The substantive nature of citations was also considered (e.g., methodological engagement vs. contextual acknowledgment). Citation relationships between the included outputs were analyzed using the edge list method (46) and visualized using network graphs with the Cytoscape network visualization tool (47).

Policy impact was assessed through Altmetric data, capturing citations in formal policy documents and grey literature, as well as broader online engagement beyond traditional academic metrics. Finally, authors’ keywords were compared with AI-extracted key terms from full texts using the Dimensions.AI algorithm to examine conceptual emphasis and identify potential blind spots.

## 4 Results

This section profiles the twelve included outputs, followed by findings from the bibliometric analysis, narrative synthesis, and impact assessment.

### 4.1 Included outputs

**Figure 1:**
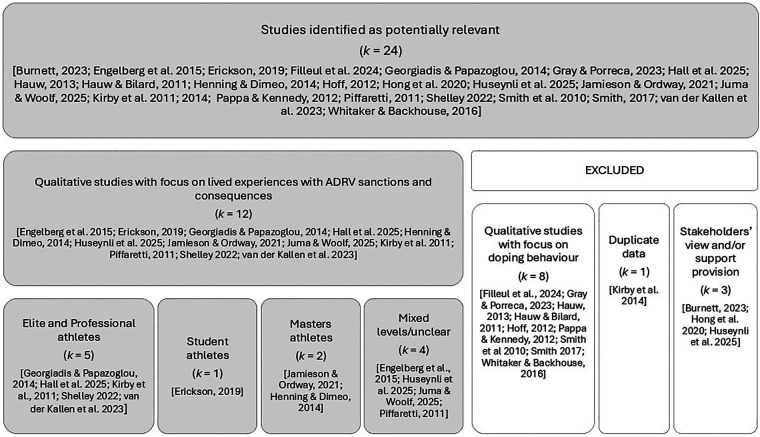
Schematic overview of the selection and inclusion process of studies on sanctioned athletes' experiences, showing identified potential (*k* = 24) and retained outputs (*k* = 12).

Twenty-four outputs were identified as having potential relevance and compared against the inclusion and exclusion criteria (Figure 1). Following this process, twelve outputs were included in the final corpus (Table 1). Three of the twelve outputs were co-authored or supervised by members of the authorship group (25, 41, 42).

The corpus comprises nine peer-reviewed articles (24, 25, 35–40, 42), one PhD thesis chapter (41), one peer-reviewed book chapter (32), and one publicly available research report (23) (see Supplementary Table 3.1). The nine peer-reviewed articles were published in Performance Enhancement & Health (k = 3), Journal of Clinical Sport Psychology (k = 2), Journal of Physical Education and Sport (k = 1), Drugs: Education, Prevention and Policy (k = 1), Sport Management Review (k = 1), and International Journal of Sport Policy and Politics (k = 1).

Methodologically, nine outputs used individual interviews to capture the perspectives of athletes (23–25, 35, 38–42). Three outputs adopted case-based approaches: one was framed as an academically oriented case study published in a peer-reviewed journal (36), one was presented as a case study within a peer-reviewed book chapter (32), and one reported the perspective of a single athlete situated within a broader dataset (37).

Temporally, no outputs were published during the first iteration of the WADC (2004–2008). Four outputs (33.3%) were published during the second iteration (2009–2014): Kirby et al. (40), Piffaretti (23), Georgiadis & Papazoglou (24), and Henning & Dimeo (37). Two outputs (16.7%) were published during the third iteration (2015–2020): Engelberg et al. (35) and Erickson (36). Six outputs (50%) were published during the fourth iteration (2021–2025): Jamieson & Ordway (32), Shelley (41), van der Kallen et al. (42), Juma & Woolf (39), Hall et al. (25), and Huseynli et al. (38).

### 4.2 Quality assessment

With a maximum possible quality score of 10, overall quality assessment scores of the individual raters ranged from 2.5 to 10. Across all raters, the mean quality assessment scores ranged from 4.67 (±0.8) to 10 (±0.0) (Supplementary Table 2.2).

The initial Inter Rater Reliability Score, based on the Fleiss’ Kappa value, was moderate with kappa = 0.41 [0.36–0.46] (48). Following discussion and revision, Fleiss’ Kappa value rose to 0.67 [0.60–0.72], which is interpreted as substantial (48). Subsequent analysis of weighted Cohen`s Kappa Analyses between the individual raters following the discussion were between moderate with kappa = 0.54 [0.33–0.75] and near perfect with kappa = 0.96 [0.92–1.00] (49); (Supplementary Table 2.3).

Generally, the authorship group identified congruence between research methodologies, output objectives, and data analysis. The voices of the athletes were also considered to be well represented throughout the analyzed outputs. However, best practices were not observed across the corpus regarding the reporting of the philosophical positioning and in situating the researchers culturally and/or theoretically, including how these factors may have influenced the research. During the quality assessment process, papers receiving lower scores were typically authored by individuals who had initially trained or worked in fields where qualitative methods are less commonly used.

### 4.3 Participant characteristics

Across the 12 outputs, the perspectives of 49 athletes were presented; however, the inclusion of the same athlete in more than one output could not be ruled out. Seven outputs presented the perspectives of athletes who had experienced the WADC sanctioning process in isolation (23–25, 32, 36, 39, 42), accounting for 34 athletes. The remaining five outputs included these athletes as part of broader samples (35, 37, 38, 40, 41), accounting for 15 athletes. Regarding the sanctioning status of participants, Engelberg et al. (35) included the perspectives of two athletes who had been “detected”, although it was not confirmed whether they were ultimately sanctioned. Henning and Dimeo (37) included one athlete who was indirectly exposed to the Article 10 sanctioning process through an unsuccessful Therapeutic Use Exemption (TUE) application but was never formally sanctioned. Huseynli et al., (38, p. 7) included athletes who had “undergone anti-doping proceedings in the last five years (pre-hearing, hearing, and/or appeal) or were serving a ban for having committed an ADRV”. In the remaining outputs, all athletes were explicitly reported as having been sanctioned for an Anti-Doping Rule Violation under the WADC.

#### 4.3.1 Gender and age

Of the 49 athletes, 32 were identified as male (65.31%), 6 as female (12.24%), and 11 had no gender information provided (22.45%). With respect to gender representation across outputs, six outputs presented the perspectives of male athletes in isolation (23, 32, 36, 37, 40, 41). Three included both male and female athletes (24, 38, 39). Three outputs provided no gender information (25, 35, 42).

Age was reported for 17 athletes (34.69%) across three outputs. Ages at sanctioning ranged from 22 to 35 years (mean 27.6 ± 3.95) in Piffaretti (23), from 22 to 29 years in Georgiadis and Papazoglou (24), and Henning and Dimeo (37) report the case of a 58-year-old athlete. Age was not reported across the remaining nine outputs (25, 32, 35, 36, 38–42).

#### 4.3.2 Geographic representation

Six outputs presented single-country samples: Australia (35), the United Kingdom (25), New Zealand (32), Austria (42), the United States (37), and Kenya (39), accounting for 20 athletes (40.81%). Piffaretti (23) included a multi-national European sample recruited from four linguistic regions: Italy, German Switzerland, French Switzerland, and Spain (11 athletes, 22.45%). Shelley (41) included one athlete from Northern Africa who had lived in New York for 15 years; the nationality of the other was unspecified. Geographic information was not provided for the remaining 16 athletes (32.65%) (24, 36, 38, 40).

#### 4.3.3 Sports and competitive levels

Athletes were recruited from a variety of individual and team sports across multiple competitive levels. 20 athletes (40.82%) competed in individual sports including running and athletics [(39) -10 athletes (41); -2 athletes], cycling [(37) -1 athlete (23); -4 athletes], surfing [(32) -1 athlete], taekwondo [(23) -1 athlete], and artistic gymnastics [(23) -1 athlete]. An additional 5 athletes (10.20%) competed in unspecified individual sports (24). Team sports were reported for 9 athletes (18.37%), including rugby union or rugby league (25) -2 athletes, American football (36) -1 athlete (23); -1 athletes, football/soccer (23) -1 athlete, basketball (23) -1 athlete, and bobsleigh (23) -3 athletes. Sporting information was not specified for 16 athletes (32.65%) across four outputs (35, 38, 40, 42).

The majority of athletes whose competitive levels were specified competed professionally, internationally, or at Olympic level (23 athletes; 46.94%). Eleven athletes (22.45%) competed at national or international level (38, 42), two at Masters level [(32, 37)–4.08%] and one student-athlete [(36)–2.04%]. Competitive levels were not reported for 12 athletes (24.49%) across two outputs (35, 39).

#### 4.3.4 Substances and violation types

Most athletes were sanctioned for prohibited substance-related violations. Performance-enhancing substances were specified for 11 athletes (22.45%), including erythropoietin 2 athletes (41); 1 athlete (23); testosterone 1 athlete (37); 3 athletes (23); clenbuterol and dianabol 1 athlete (32); boldenone 1 athlete (23); oxilophrine 1 athlete (23); and chetamine 1 athlete (23). The five athletes in Georgiadis and Papazoglou (24) were sanctioned for the presence of either methyltrienolone (metribolone) or testosterone; however, it is unclear which substance each athlete tested positive for, or whether any tested positive for both. Juma & Woolf (39) report sanctions by substance category rather than specific substances: Hormone and Metabolic Modulator (1 athlete), Anabolic Agent (5 athletes), Glucocorticoid (1 athlete), and Beta-2 Agonist (3 athletes). Recreational substances accounted for three sanctions [6.12%; hashish—2 athletes; cocaine—1 athlete; (23)], and one athlete was sanctioned for attempted use and trafficking (23). A further eight athletes (16.33%) were sanctioned for substance-related violations with unspecified substances (25, 35, 36, 40). In 11 cases (22.45%), it was unclear whether violations were substance-related or involved other anti-doping rule violations (42)–4 athletes; (38)–7 athletes.

#### 4.3.5 Sanction duration and WADC code

Sanction duration was specified for 26 athletes (53.06%). Twelve athletes (24.49%) were sanctioned for 24 months [8 athletes; (23); 2 athletes; (40); 2 athletes; (39)], seven athletes (14.29%) received four-year sanctions [1 athlete; (41); 5 athletes; (39); 1 athlete; (23)], three athletes (6.12%) received three-year sanctions (39), two athletes (4.08%) received 18-month sanctions 1 athlete; (41); [1 athlete; (23), and one athlete (2.04%) received a 12-month sanction (23)]. One athlete (2.04%) received a lifetime ban for a second offense (40). Erickson (36) reported one athlete serving at least two years, though the total length was unclear (2.04%). The remaining 22 athletes (44.90%) had no sanction duration specified. One athlete in Piffaretti (23) received two sanctions.

WADC version information was provided for 11 athletes (22.45%) across two outputs. Jamieson and Ordway (32) referenced the 2015 Code, while Juma & Woolf (39) included 5 athletes sanctioned under the 2015 Code and 5 under the 2021 Code. The remaining 38 athletes (77.55%) had no WADC information reported.

#### 4.3.6 Sanction status and return to sport

Sanction status at the time of data collection was reported for 25 athletes (51.02%). Seven athletes were still serving their sanctions at the time of discussion by the original authors (36, 39, 41). One athlete (2.04%) had completed their sanction but had not returned to sport (41). Three athletes (6.12%) had returned to sport (39), and two athletes (4.08%) had returned but subsequently retired (39). Three athletes (6.12%) from Kirby et al. (40) were not competing at the time of interview. Van der Kallen et al. (42) included four former athletes (8.16%) for whom the timing relative to sanctions was unclear. Georgiadis & Papazoglou (24) interviewed five athletes (10.20%) who were 8–10 months post-ban, without clarifying whether sanctions were completed. One athlete in Jamieson & Ordway (32) received a formal two-year sanction, backdated by one year. The remaining 23 athletes (46.94%) had no sanction status information reported.

#### 4.3.7 Admission of guilt

Admission or denial of violations was reported for 21 athletes (42.86%). Piffaretti (23) classified five athletes as accidental consumption, five as regular consumption, and one as attempted use. Van der Kallen et al. (42) included four athletes (8.16%) who admitted intentional violations, while the five athletes (10.20%) who participated in Georgiadis & Papazoglou (24) all maintained innocence. Athlete XYZ was reported to be unaware that he was bound by the obligations set out in the WADC (32). The remaining 28 athletes (57.14%) had no information on admission or denial of violations.

### 4.4 Athletes’ subjective accounts of experiencing and enduring sanctions under the WADC

The narrative synthesis suggests that the consequences associated with WADC sanctions extend beyond sporting implications and can emerge prior to the imposition of a formal sanction, fundamentally reshaping athletes’ lives. The following analysis maps these experiences, ranging from the initial uncertainty of investigations and acute psychological distress to the longer-term effects of social stigmatization, institutional abandonment, and the disruption of athletic identity.

#### 4.4.1 Impacted without a confirmed sanction

The athletes included in Engelberg et al. (35), Henning and Dimeo (37) and Huseynli et al. (38) were either not sanctioned or had an unclear sanctioning status, yet the experiences reported in these outputs were thematically comparable to those of athletes who had been explicitly sanctioned. These similarities support the inclusion of these perspectives within the wider sample and indicate that comparable consequences of the WADC sanctioning process can arise in contexts where the status of a formal sanction remains uncertain.

#### 4.4.2 Physical, psychological and emotional distress

Athletes across multiple outputs reported negative physical, psychological, and emotional effects after being exposed to the ADRV sanctioning process (23–25, 36, 38–42). These effects were experienced during the sanctioning process, throughout the period of ineligibility, and following the completion of the formal sanction. Focusing on the emotional impact of sanctioning, Piffaretti (23, p. 39) presented a quantified range of emotional responses to being sanctioned:

The most frequently mentioned were anger (reported by 72.7% of the sample), sadness (36.4%), and disappointment (27.3%). Other emotions included hope, denial, confusion, existential fear, regret, incomprehension, guilt for the athletes’ environment, loss of self-esteem, and frustration (perception of injustice).

Beyond the quantitative reporting of emotional ranges, athletes' testimonies illuminate how these responses are experienced in practice: “I cried endlessly. For three months, there was not a day without tears..It felt like I had 100 kilos right on my chest” (24, p. 440).

The testimonies reveal a trajectory of emotional impacts experienced by athletes throughout the sanctioning process, beginning with feelings of “shock” in response to the “intensity” of ADRV investigations (38, p. 8) and continuing through to longer-term consequences:

After the initial anger and incomprehension came a feeling of emptiness, followed by despair, which was linked to the negative consequences of the sanction on potential career opportunities as a coach, provoking a complete psychological breakdown, which required hospitalisation in a psychiatric clinic and continued psychotherapeutic follow-up until the moment of the interview, namely 1½ years later (23, p. 39).

Juma and Woolf (39, p. 5) reported that sanctions could lead to changes in athlete behavior. Two athletes were described as increasing their alcohol intake following a sanction, with one case presented in further detail: “Gathenge, turned to alcohol, saying, “I stayed alone. I did not want anybody in the house. I was just staying alone, drinking, and then trying to figure out things””. In more severe cases, behavioral responses lead to suicidal ideations being described by multiple athletes including one case where an athlete “attempted suicide after months of isolation. She recalled, “In the third month, I felt like things were at a climax, so I tried committing suicide””.

Alongside the negative mental health consequences, decreases in physical health were reported in two outputs (39, 42). Athletes describing cases of insomnia and oversleeping; one athlete recounted:

I got ulcers immediately after the sanction. [..] I had a very bad headache. I was taken to the hospital, and I was told it was because I was thinking too much. The doctor told me that the headaches and ulcers were because of too much acidity in the body. (39, p. 5)

Similar experiences were described in van der Kallen et al. (42), with two athletes describing physical symptoms. One athlete described: “After some time I was prone [to illness], I picked up something everywhere”. Another linked the enforced change in routine with changes to their physical health: “My physical well-being suffered because of a lack of training”.

These negative impacts were described to extend beyond the implicated athletes to their families (25, 39). For example, a rugby player explained: “The ban didn't really have that much of an effect on me, but it really affected my family. It put a massive strain on my mum. My family struggled with it more than I did” (25, p. 5). Within Juma & Woolf (39, p. 6) cases of parental separation and the indirect impact of increased alcohol consumption linked to a sanction had on a child: “his son was distressed by his drinking and told him, “If you die today because of alcohol or do something bad, look, now the family is breaking””.

#### 4.4.3 Stigmatization and social isolation

Feelings of social isolation and stigmatization were described by several athletes (23–25, 36, 39, 41, 42). While related to the emotional and behavioral responses described, the in-depth and consistent accounts of sanctioned athletes feeling stigmatized and engaging in self-initiated isolation reflect a specific aspect of the lived experience of athletes sanctioned under the WADC.

Stigmatization was understood to refer to a social process through which individuals are discredited or labelled due to an attribute or event that places them outside accepted norms. Through the testimonies of athletes, stigma was evidenced through reputational damage, public condemnation, and exclusion from communities. For example, a bobsledder explained: “Now I have an indelible stamp which is still a problem today to get a new job” (23, p. 39).

Other accounts described the social rejection that followed sanctioning, drawing attention to the shift from being celebrated to becoming ostracized:

When you realize [you went] from the point of being number one and everybody wanting to stand next to you to suddenly finding yourself at the bottom, with everyone turning their back on you..these are the really hard times. (24, p. 9)

Sensationalized media coverage was described as a contributing factor that intensified the impact of sanctioning (23, 24, 36, 41). One athlete emphasized how media distortion shaped the social consequences of sanctioning: “The reporter in a broadcast is willing to distort and tear everything apart in order to sell his story” (24, p. 440).

Athletes also reflected on how online exposure intensified reputational damage and removed any possibility of distancing themselves from the sanction. For example, a student-athlete explained:

Next thing I know, it's all over Facebook and all my former teammates are messaging me about it, some immediate family are messaging me about it and what do you say to people? They don't understand what happened and I don't really want to talk about it with everybody… You can Google my name and it will blow up with news on my sanction (36, p. 225–226).

For some athletes, stigmatization was experienced as reputational harm and continuing condemnation, extending beyond the sporting context:

My crime was in the sports rules, so punish me within the sports rules..I'll serve my ban, but don't try to ruin my whole reputation as a person… You cannot make people forgive what you've done… the only thing that would be enough for some people is to kill yourself (41, p. 91).

These experiences were linked to athletes' decisions to withdraw from their sporting communities and daily routines as a form of self-protection (24, 25, 36, 41). This withdrawal extended beyond competitions and training spaces, sometimes including efforts to avoid reminders of their former athletic lives:

I don't use social media but if I go on my email and see any running stuff, I just delete it in case it is more abuse. Imagine how much I have loved running, I'm 33 years old and I've always read constantly into running, and now I don't want to even open an email. When I see any running people when I'm going to work, I just turn down a different street because I don't want to see them (41, p. 91).

The same athlete further explained the reasoning behind this self-isolation:

Don't try to make me suffer every day… don't try to make me out to be the worst person in the world, because that's what people have done, and at some point in the last few months, I started hating people so much, and even the running community (41, pp. 90–91).

#### 4.4.4 Institutional abandonment and perceived procedural injustice

Institutional abandonment and procedural unfairness were recurrent concerns among athletes (23–25, 32, 35–39, 41). Huseynli et al. (38) was the only study in the corpus to explicitly examine athletes’ procedural perceptions of the sanctioning process in isolation from other aspects of the sanctioning experience, revealing complex and often contradictory reflections. Four of the seven participants characterized their overall experience as positive, noting that they felt listened to and that the proceedings were clearly explained, yet five participants simultaneously described negative aspects, particularly the “intensity” (p. 7) of investigations and perceiving the process as biased, feeling they were presumed guilty before being able to prove their innocence. Despite some positive remarks, typically a sense of dissatisfaction was presented, with athletes feeling they “frequently lacked crucial information or guidance” (p. 7) and felt “ill-prepared for the hearing” (p. 8).

This tension between positive and negative evaluations extended to perceptions of fairness, which revealed notable contradictions particularly regarding the pre-hearing phase, where four athletes initially described this stage as “fair” (38, p. 8) yet six subsequently indicated they were treated unfairly during this same phase. Fairness perceptions became more consistently negative in relation to the adjudication stage, with none of the athletes describing this phase as fair with an athlete describing feeling “small, insignificant and powerless” (p. 8) and “unable to do anything to contest or overturn” (p. 8) their sanctions. Hearing panels were uniformly described as competent and impartial, with one athlete describing the panel as seeming “human” and that “they seemed to listen and understand” (p. 8). Legitimacy perceptions similarly varied, with two athletes accepting the “strictness” (p. 7) of proceedings as a legitimate reflection of anti-doping enforcement while three perceived the overall process as “illegitimate” (p. 7), with one explaining they “did not understand the process enough to feel protected” (p. 7) and another stating that “illegitimate means and methods of gathering information were used” (p. 7). These accounts demonstrate that athletes’ evaluations of sanctioning were shaped by institutional practices, communication quality, and information provision, producing experiences that were contradictory, mixed, and highly context dependent.

Perceived injustice around institutional support and procedural fairness was compounded by athletes describing a lack of awareness of their responsibilities under the WADC which further contributed to this sense of exposure:

The athlete is the scapegoat, and the one who has to pay; after much thought I think that something was wrong with the substances that we were receiving and that those who provided the vitamins to us did not know about it. At any case, I was the one who took the blame (24, p. 440).

The perceived lack of differentiation between intentional and inadvertent violations was also described as a source of feelings of injustice:

A11 goes on to say that the sanction he got was unfair: The thought that he was punished with the same kind of sanction than other athletes who had admitted an intention to break the anti-doping regulations frustrated him very much (23, p. 38).

This issue extends beyond elite sport to Masters and amateur athletes. One athlete who required testosterone treatment but was denied a Therapeutic Use Exemption described the impact of the strict liability principle on their experience:

They're treating us like 20-year-old Olympians. Something that's considered a performance-enhancing drug for an 18-year-old may be a necessary life-saving medication for a senior athlete. I think it's very unfair (37, pp. 405–406).

Following the imposition of a sanction, feelings of institutional abandonment were described (36, 39):

I didn't get any help from my university other than them making it clear that they didn't want me to talk about anything in the media. They were super nice with me and made it seem like they had my back, but afterward they basically said, “you can't come back to the complex anymore” (36, p. 225)

From a Kenyan context, several athletes reported difficulties in contacting Athletics Kenya (AK) following a sanction. Some athletes received no response when they sought assistance, while others reported choosing not to contact AK anticipating that their concerns would be dismissed or judged. These experiences reflect a broader sense of isolation and lack of institutional support for athletes navigating the sanctioning process. As one athlete summarized, “When you are sanctioned, you are alone. I don't think AK will listen to you” (39, p. 6).

#### 4.4.5 Disruption to career and identity

Whilst career disruption is an inevitable consequence of an ADRV sanction with athletes facing a period of ineligibility under Article 10 of the WADC, athletes described disruption that extended beyond sporting contexts and their sanction, towards a broader loss of self-identity (24, 25, 36, 37, 39, 41).

For example, some athletes experienced job disruption linked to the wider consequences of their sanctioning. In Kenya, athletes who were employed by the police or military through sports programs experienced forced redeployment following a sanction, which required relocation creating unplanned financial burdens such as breaking lease agreements and securing new accommodation (39). Georgiadis & Papazoglou (24, p. 439) corroborated this pattern, noting that athletes were “asked to leave their place of residence”, as elite housing provided by the sporting federation was withdrawn. In some cases, this disruption resulted in job loss: “I was working at the university fitness centre at the time so not only was I banned from doing anything with the football team, but I was immediately fired from my job” (36, p. 225).

Logistical and occupational changes compounded the immediate loss of status and income. Financial strain affected athletes and those around them. For example, one athlete described: “For the first time in my life I had to struggle to make ends meet—the repayments were tremendous, and attorney fees and legal expenses were very high” (42, p. 6).

Beyond material consequences, athletes reflected on the disruption of their identity. One athlete explained: “Back in the old days, it was different. I was certain that when I would stop competing there would be some open doors waiting. But after that (the positive doping sample), I only see closed doors everywhere” (24, p. 440). A different athlete from the same study highlighted the resulting disorientation: “I have not learned to love anything else in my life, I do not know where else I'm good at..It seems strange to me not being able to engage myself with what I know I'm good at” (24, p. 440). Similarly, an athlete described the identity disruption of being excluded from competition: “I still cannot believe that I do not compete. I am away from my objectives; I am far from my goals, out of life. I'm somewhere else; I'm not an athlete anymore” (24, p. 440).

#### 4.4.6 Relief in ruin

A minority of athletes described ADRV sanctioning as having positive impacts on their lives. For some, a sanction prompted a sense of relief and provided an opportunity to disengage from the pressures associated with high-performance sport:

It's strange, but when I received the sanction, I felt a sense of peace. I had been caught in a cycle of frustration and dissatisfaction with the sport. The ban gave me an excuse to walk away, to finally escape all the expectations and the constant stress that had been building up. It was like the decision was taken out of my hands, and I could just breathe again (23, p. 45).

A rugby player described a similar experience:

At first, I was in shock, but then I realised something. I was actually relieved. It was like a door closed, but in a good way. I had been miserable for so long, but I had no way of leaving on my own terms. The sanction gave me an out, a chance to reset and look at my life from a new perspective. It was probably the best thing that happened to me, even though it was hard to admit at the time (25, p. 7).

Alongside the emotional relief, athletes reported that sanctioning enabled the identification of new opportunities. Juma & Woolf (39) documented several of these cases, with athletes returning to education, and reporting career development opportunities.

### 4.5 Challenges in participant recruitment

Multiple outputs reported challenges relating to participant recruitment (23–25, 35, 39, 40, 42). Beyond the reporting of challenges, Juma & Woolf (39) and Piffaretti (23) outlined the specific barriers encountered. These included relevant participants not having the time to participate (23, 39), expressing a lack of interest (23), and being unreachable (23).

### 4.6 Bibliometric analyses

To complement the narrative synthesis and deepen understanding of the research landscape on athletes sanctioned under the WADC, this section presents findings from a bibliometric analysis of the included outputs. This analysis offers a meta-level perspective on the field's development, research patterns, and structural gaps. It maps publication trends over successive WADC iterations, compares author-supplied keywords with AI-extracted terms from Dimensions.AI to identify thematic emphases and conceptual divergences, and explores the disciplinary positioning of contributing authors. The section also evaluates the degree of scholarly collaboration through network analysis and assesses academic and policy impact via citation metrics and qualitative citation patterns. Together, these insights contextualize the field's epistemic structure and signal opportunities for future research development.

#### 4.6.1 Authors’ keywords and auto-generated key terms

Areas of overlap and divergence between author-generated keywords and those extracted by the Dimensions.AI algorithm were identified (Supplementary Table 3.1). Author-generated terms tended to foreground athletes’ lived experiences and the micro-contexts of specific sports, reflecting qualitative methodologies and psychosocial constructs such as moral reasoning, identity, and coping. Keywords like “lived experiences”, “critical incidents,” and “social identity” point to narratives where athletes described the emotional toll of sanctions and the complexity of their decisions. For instance, one cyclist spoke of the moment his “whole world collapsed” after receiving a ban (23, p. 19), while a student-athlete emphasized that “behind every ban is a human story—injury, pressure, and choices that spiral” (36, p. 7). These perspectives underscore the human dimension embedded in author-selected terms. In contrast, Dimensions.AI keywords were more closely aligned with macro-level concerns of policy, governance, and institutional frameworks, emphasizing procedural fairness, legitimacy, and rights protection. This orientation mirrors athletes’ perceptions of systemic rigidity, as reflected in comments such as feeling that “he process was a punishment machine, no one cared about context” (23, p. 38) or that “due process didn't feel like that; it felt like a verdict before a hearing” (37, p. 163). Such remarks highlight the disconnect between regulatory ideals and lived realities.

Despite these differences, both sets converged on core themes such as doping, anti-doping policy, and sanctioned athletes, alongside references to psychological dimensions, health outcomes, and qualitative approaches. This shared focus reflects the duality of doping research, its regulatory imperatives and its human consequences. Athletes’ testimonies reinforce this intersection, noting that “everyone around me acted like it was normal” (23, p. 20) and that “the hardest part wasn't the ban, it was losing my identity as an athlete” (40, p. 212).

#### 4.6.3 Authors' disciplinary backgrounds

Among the 34 author affiliations, ten (30%) are situated in psychology-related disciplines, including clinical psychology, sport and exercise psychology, sport psychology, and general psychology. Eight authors (21%) are based in sports medicine or sports science, encompassing biochemistry, kinesiology, exercise and sport, and exercise science. Public or applied health fields account for three entries (9%). The remaining 12 authors (36%) represent diverse disciplines, including sport management, sports business, education, law and ethics, sociology, and sport history. The disciplinary background of one author could not be identified.

#### 4.6.4 Researcher collaboration

**Figure 2:**
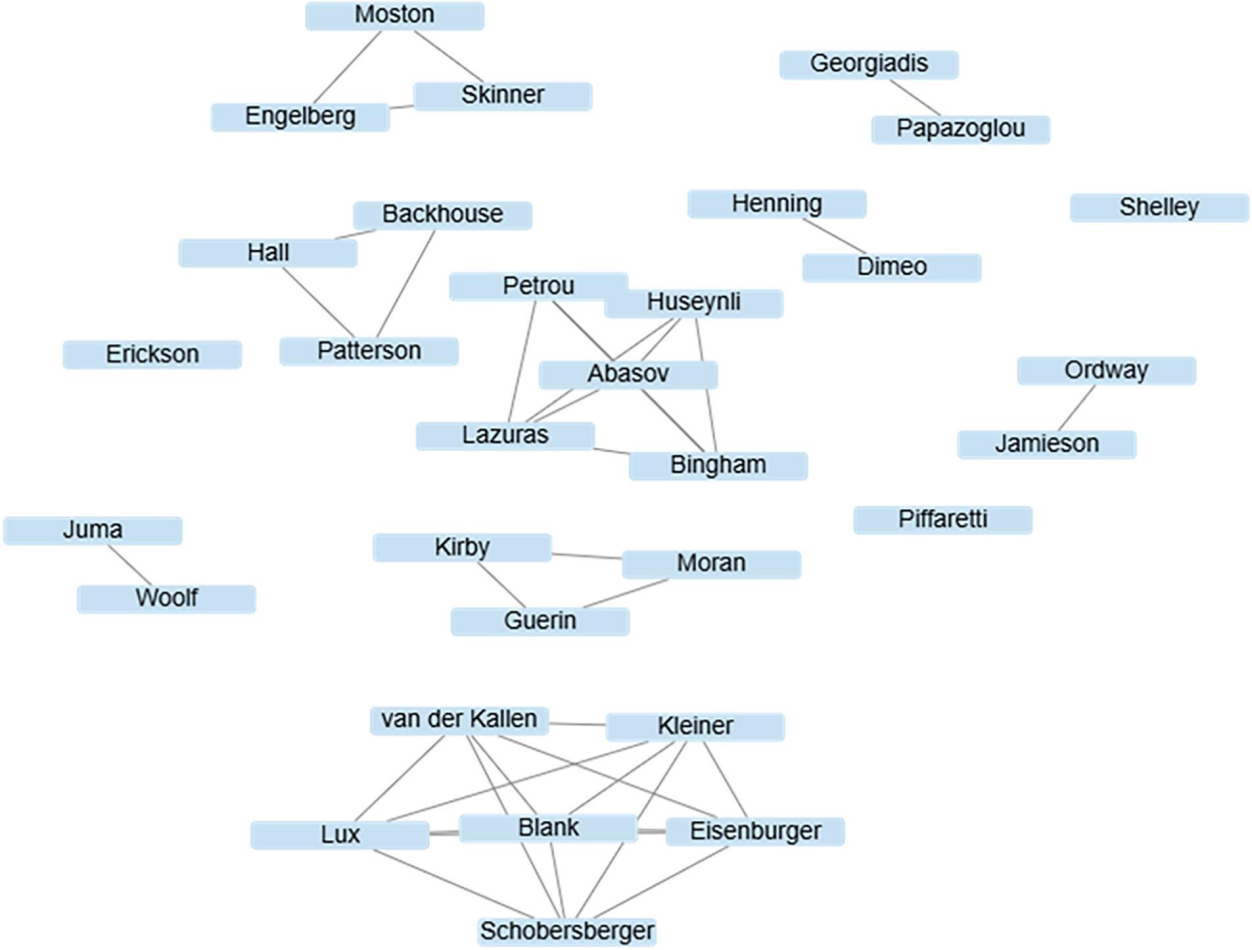
Co-authorship network illustrating collaborative relationships among authors of included studies on sanctioned athletes' experiences (*k* = 12).

The co-authorship network analysis reveals a highly fragmented structure with minimal interconnectivity among research groups (Figure 2). Two clear patterns emerged. First, collaboration is largely confined within small, isolated clusters. Most groups consist of two to four authors who co-published a single output, with no links to other groups. Second, there is an absence of cross-group collaboration. No bridging nodes or connecting ties were observed between clusters, indicating that research on sanctioned athletes has evolved in silos rather than through integrated, multi-team efforts.

### 4.7 Tracing intellectual connections: how studies build on each other

**Figure 3:**
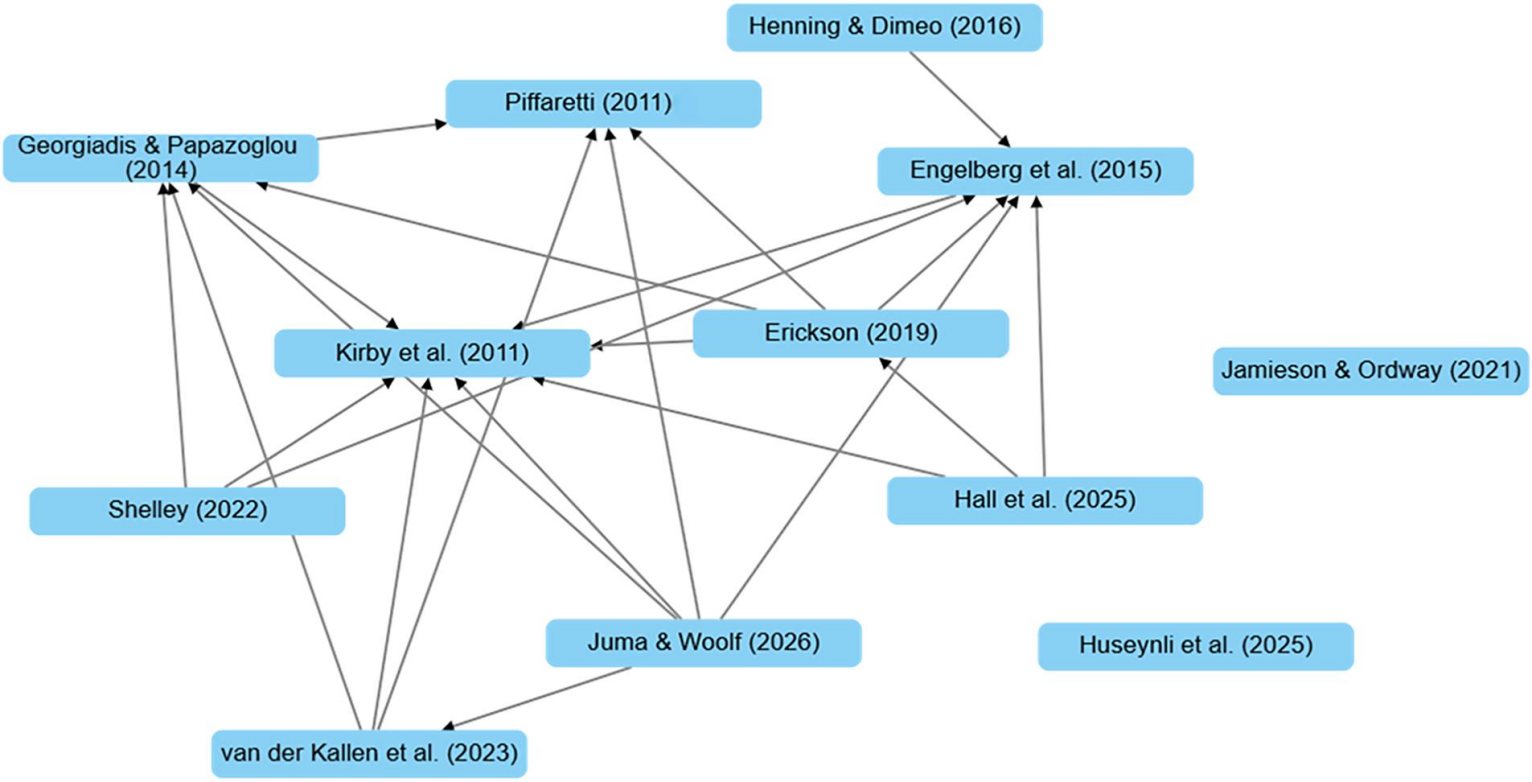
Citation network illustrating within-corpus citation patterns among the included studies on sanctioned athletes' experiences (*k* = 12).

Figure 3 illustrates the citation relationships among studies included in this review, revealing a moderately interconnected but still loosely structured network. Unlike the fragmented co-authorship pattern (Figure 2), citation links indicate some intellectual continuity across time and research groups. Overall, the citation network suggests partial integration of knowledge with a few seminal studies providing foundation for researching sanctioned athletes’ experiences. Several early works serve as central nodes in the network. Kirby et al. (40) and Piffaretti (23) appear as key anchors, receiving multiple incoming links from later studies such as Erickson (36), Hall et al. (25), and Juma & Woolf (39). These connections suggest that foundational qualitative research on sanctioned athletes and psychological determinants continues to inform subsequent scholarship. Similarly, Engelberg et al. (35) occupies a bridging position, cited by both earlier and more recent outputs, including Henning & Dimeo (37) and Hall et al. (25), reflecting its relevance to policy and deterrence frameworks.

Despite these hubs, the network remains relatively sparse. Clusters such as Georgiadis & Papazoglou (24) and Shelley (41) show multiple citation ties, yet there are notable isolates like Jamieson & Ordway (32) and Huseynli et al. (38), which have no visible outbound or inbound links within this topic set. This pattern indicates that while some studies build on prior work, others emerge independently, limiting cumulative theory development. The absence of dense cross-referencing points to the need for greater synthesis and studies offering conceptual scaffolding.

### 4.8 Impact

This section presents an integrated assessment of the included outputs, using data from Dimensions.AI, Scopus, and Clarivate (Web of Science), to identify patterns in the scale and disciplinary reach of citation impact.

Studies examining behavioral, ethical, or performance-related dimensions of sport and health show measurable academic impact and interdisciplinary reach. Kirby et al. (40) and Engelberg et al. (35) each exceed 85 citations and rank in the top percentiles for field-weighted citation impact and field citation ratios (Supplementary Tables 4.1, 4.2). Both studies are cited across multiple disciplines, including psychology, sociology, pharmacology, and sport sciences (Supplementary Table 4.3). Altmetric data indicate broader visibility, with Engelberg et al. (35) appearing in policy documents and receiving diverse online engagement. Erickson (36) and van der Kallen et al. (42) show lower citation counts but remain visible through digital platforms. Recent outputs, including Hall et al. (25) and Huseynli et al. (38), have limited citation data but demonstrate early online attention.

Citation network analysis (Figure 3) indicates a moderately interconnected field. Kirby et al. (40) and Engelberg et al. (35) serve as central nodes, with Kirby et al. (40) exhibiting the highest in-degree centrality. Juma & Woolf (39) and Erickson (36) show higher out-degree centrality, reflecting engagement with prior studies. Qualitative citation content analysis (Supplementary Table 4.4) shows variation in citation usage: some references are embedded in methods, discussion, or research rationale, whereas others serve primarily as acknowledgements. For example, van der Kallen et al. (42) and Juma & Woolf (39) cite Kirby et al. (40) and Piffaretti (23) to support research design and findings, whereas Erickson (36) and Shelley (41) cite these studies primarily for contextual background.

In terms of policy impact, past research had little documented traction. Engelberg et al. (35) was the only output identified as being cited in formal policy documents, appearing in a European Commission report on the use of anabolic steroids and human growth hormones in sport (Supplementary Table 4.3). No other outputs were found to have direct citations in policy literature. However, this limited visibility does not necessarily reflect the full extent of influence. Research in this area may have informed policy thinking or practice through informal channels (e.g., expert consultations, workshops, or internal reports) that are not captured through bibliometric tracking. While such pathways to impact or at least influence are likely, their impact remains undocumented and difficult to systematically assess within the scope of this analysis.

## 5 Discussion

This review presents an exploratory synthesis of peer-reviewed outputs offering insights from athletes who have experienced the WADC sanctioning process and how these experiences were interpreted by the primary authors. Twelve outputs were included in the final review, published over a fourteen-year period, and analyzed using three methodological approaches: bibliometric analysis, narrative synthesis, and impact assessment. Collectively, these outputs and analyses provide a foundational understanding of an area that remains under-researched and under-theorized.

The bibliometric analysis highlights broader trends in anti-doping social science research, showing a field that remains structurally underdeveloped, lacking the institutional depth and continuity typical of more mature research areas (50, 51). This under-institutionalization reflects a marginal status within sport policy research agendas (52–54). Outputs are largely produced by dispersed, de facto collaborations, often arising from student-led or single-project initiatives, with many contributions originating from postgraduate theses [e.g., (39, 41, 42, 55)]. While the quality of these outputs is not in question, the isolated nature of this research limits opportunities for theoretical development, longitudinal tracking, and comparative work (56), leaving the field vulnerable to knowledge silos and slow conceptual progress. The bibliometric findings, together with prior literature (26), suggest that the current configuration of research into WADC sanctions constrains the extent to which findings meaningfully inform policy development.

These structural challenges do not indicate a lack of importance. The narrative synthesis demonstrates that athletes exposed to the WADC sanctioning process experience consequences that extend beyond the sporting domain, sometimes with life-changing or life-threatening effects. Reported impacts include physical illness, psychological trauma, financial stress, reputational damage, and disrupted career trajectories. These outcomes resonate with established literature on the psychosocial toll of enforced career disruption and stigmatization in elite sport. Sanctions often act as involuntary, identity-threatening life transitions, reflecting research on athletic identity foreclosure and the psychological instability following sudden disengagement from sport (57, 58). Reputational damage and social exclusion also echo Goffman's (1963) concept of stigma and the “spoiled identity,” particularly where public perception frames athletes as guilty or deviant (52, 59). Financial hardship, including lost sponsorships, legal costs, and disrupted careers, is exacerbated by limited safety nets during and after sanctioning (60). While many athletes reported profound harm, some described sanctions as catalysts for positive change, offering relief from sporting pressures, opportunities for personal growth, or pathways to sustainable careers beyond elite sport. These adaptive responses resemble post-traumatic growth described in crisis and coping literature (61), particularly when dual career planning or educational support is available (62, 63). Together, these accounts underscore that doping sanctions are not merely regulatory mechanisms but disruptive social events that fracture or reshape identity, career, and social standing, making legitimacy concerns tangible and embodied rather than abstract. Analysis of athlete perspectives suggests that the standards of legitimacy outlined by Woolway et al. (2) are not fully realized, with both macro-level policy and micro-level implementation practices shaping perceptions of fairness, proportionality, and institutional trust.

At the macro-policy level, decisions such as the application of strict liability and minimal differentiation based on intent contributed to perceptions of unfairness, challenging both procedural and performance legitimacy. Across multiple WADC iterations, athletes reported consistent experiences, indicating that successive procedural revisions have not substantially altered perceptions of fairness. These recurring harms and procedural concerns reinforce the “wicked problem” nature of doping governance (64, 65), where policy effects are only fully apparent once athletes encounter them. The absence of clearly articulated objectives beyond deterrence complicates assessments of proportionality and raises questions about whether harms represent unintended consequences or acceptable costs of enforcement. These concerns align with broader academic commentaries questioning procedural legitimacy in anti-doping governance (2, 66, 67). Strict liability, in particular, has been contested as incompatible with fundamental principles of justice and rights-based approaches in sport (20, 68). Taken together, athlete accounts and scholarship suggest that macro-level policy raises enduring concerns for performance legitimacy, with questions of proportionality emerging in balancing deterrence against fair and proportionate sanctioning outcomes. The human cost of ADRV sanctioning, even based on limited empirical evidence, appears largely at odds with WADA's stated objectives of maintaining sport integrity and safeguarding athlete health (5).

At the micro-implementation level, experiences of institutional neglect and inconsistent procedural communication strongly shaped perceptions of anti-doping proceedings. Athletes frequently reported feeling excluded, unsupported, or inadequately informed, producing emotional distress, confusion, and enduring mistrust. These consequences align with procedural justice literature, where fairness, respect, and transparency are central to legitimacy (7, 69). Institutional responses, including disbelief, blame, and limited support, amplified psychological harm, reflecting secondary victimization (70) and epistemic injustice, which undermines athletes’ capacity to be heard and trusted (71). Unclear updates, inconsistent application of rules, and limited support reinforced feelings of exclusion and mirrored patterns in other institutional contexts, such as healthcare and criminal justice, where opaque communication undermines trust and exacerbates victimization (72, 73). These micro-level challenges were reported consistently across outputs, indicating geographical and contextual validity rather than isolated incidents.

The operationalization of the WADC sanctioning framework illustrates how macro-level decisions manifest in athletes’ lived experiences, producing a legitimacy gap across structural and interpersonal dimensions. Strict liability and limited consideration of intent appear at the micro-level as disempowerment, reinforcing asymmetrical obligations and minimal institutional reciprocity (74). These experiences compromise both procedural and performance legitimacy (75), revealing that the macro-framework cannot be fully evaluated without accounting for how it is enacted and felt. Evidence indicates that such insights rarely influence policy revisions, highlighting the adaptive deficit characteristic of wicked governance problems (26).

Addressing these challenges requires targeted interventions to strengthen the micro-implementation environment. Greater transparency, consistent communication, clear procedural updates, structured support mechanisms, and guidance on navigating the process would mitigate exclusion and disempowerment. Collaborative capacity-building among NADOs and other regime actors could address resource limitations and improve anti-doping governance (1). Aligning micro-level practices with macro-level oversight would help ensure the system operates fairly, proportionately, and legitimately, reducing stigma, isolation, and disruption. Embedding athlete welfare as a key performance indicator within WADA's oversight would further strengthen this alignment, supporting a fairer system while leveraging existing top-down regulatory structures. Reform efforts must also address fundamental questions of proportionality and legitimacy at the policy design level. Advancing understanding requires a comprehensive, evidence-informed approach drawing on multiple data sources and perspectives, alongside collaboration among researchers, athletes, anti-doping practitioners, and policymakers to co-develop practical, context-sensitive, and socially acceptable solutions. This collaboration is essential to balance deterrence with procedural justice in ways that reflect the perspectives of all sub-populations of athletes. Mechanisms should prevent unintended harms while ensuring sanctions remain consistent with the values and expectations of the athlete population, grounding legitimacy in both institutional authority and meaningful stakeholder consensus.

### 5.1 What can be done and whose responsibility is it?

The integration of findings from this study with those of Hong et al. (76), which highlighted limited organizational support for athletes sanctioned under the WADC, reinforces the presence of a definable and systemic gap in care provision for athletes who are exposed to the WADC sanctioning framework. Based on the testimonies examined in this review, this population is currently being overlooked despite the expansion of interventions aimed at enhancing athlete welfare (77, 78). Whilst the existence of this unmet need is problematic, it also offers organisations such as WADA an opportunity to develop a support system from the foundations upwards rather than amending legacy frameworks, which could pose challenges due to barriers relating to policy innovation (79–82). As part of this foundational development, there is also a need to develop an understanding of which organizations have or could inherit a responsibility for providing care for this population of athletes.

A logical starting point is the responsibility of WADA as the primary policy maker and overseer within global anti-doping governance, specifically in relation to how the aims of sanctioning are articulated and justified. Whilst the WADC (5) outlines the rationale for anti-doping regulation, provides a harmonized definition of doping, and describes how to sanction athletes, limited attention is given to clarifying the broader purposes of sanctioning, creating uncertainty regarding the intended outcomes of periods of ineligibility for those who experience them. This ambiguity persists in the latest iteration of the WADC, which comes into effect in 2027; however, anti-doping is now explicitly framed as “primarily an ethical position based on a vision of the spirit of sport” (83). Framing anti-doping in ethical terms strengthens the normative basis of sanctioning but also elevates expectations that the system attends to the wider consequences experienced by sanctioned athletes, rather than limiting its focus to rule enforcement alone. As a legitimacy-based authority (1, 2), WADA therefore carries responsibility not only for maintaining perceptions of the World Anti-Doping Program, but also for anticipating and mitigating unintended harms arising from the application of its rules (8). This includes ensuring that sanctioning practices align with principles of transparency, accountability, proportionality, and fairness (2, 7), and that the purposes of Article 10 are sufficiently articulated to those subject to it.

Whilst this review has primarily focused on athletes’ lived experiences through the lens of perceived legitimacy, WADA also carries responsibilities under the World Anti-Doping Program to uphold the rights and protections of the athletes it governs, reflecting both regulatory and legal obligations. Recent developments illustrate this dual dimension. WADA has initiated a Human Rights Impact Assessment to examine how the World Anti-Doping Program intersects with the human rights of athletes (13). Led by an independent expert, the assessment aims to identify risks and ensure that anti-doping governance aligns with internationally recognised human rights standards, complementing broader efforts to sustain legitimacy and safeguard athletes throughout the sanctioning process. Alongside this internal review, external legal scrutiny has also emerged. The Advocate General's opinion before the Court of Justice of the European Union in Case C-474/24 questioned whether the systematic publication of athletes’ names, sporting discipline, sanction length, and reasons for sanction may be disproportionate (84). The opinion pointed towards alternatives such as pseudonymized publication or more targeted disclosure to relevant bodies, balancing transparency with data protection considerations. Taken together, these developments indicate that WADC sanctioning practices are being evaluated not only in terms of perceived legitimacy, but also against legal and organizational standards, reinforcing the relevance of this issue for WADA in sustaining legitimacy and fulfilling its governance responsibilities.

Alongside WADA, signatory organizations to the WADC carry direct responsibility for the implementation of anti-doping rules in practice. NADO, International Federations, Regional Anti-Doping Organizations, and accredited laboratories are responsible for translating the WADC into operational procedures across testing, education, results management, and sanctioning. These responsibilities are exercised within diverse socio-geographic, political, organizational, and resource contexts, requiring signatories to manage variation while maintaining compliance with the standards of the World Anti-Doping Program (1, 4). As the primary point of contact between the anti-doping system and athletes, signatories are responsible not only for technical compliance, but also for sustaining legitimacy through their everyday practices (85). This includes acting transparently in testing and sanctioning procedures, maintaining accountability in decision-making processes, and applying sanctions in a proportionate and consistent manner. Signatories also hold responsibility for protecting the independence of anti-doping processes from political or organizational interference and for clearly communicating their roles, obligations, and procedural steps to athletes (86). Where capacity constraints exist, signatories are responsible for pursuing collaboration, information-sharing, and capacity-building mechanisms, including bilateral agreements, to ensure that standards are met in practice (87). Through the clear articulation and consistent enactment of these responsibilities, signatories play a central role in maintaining the legitimacy of the anti-doping regime among athletes and in shaping how the WADC is experienced in everyday sporting contexts.

Despite these responsibilities, the testimonies analyzed in this review suggest that neither WADA nor WADC signatories are well positioned to deliver the forms of pastoral or welfare-oriented support that sanctioned athletes describe as necessary. This limitation reflects not only resource constraints, but more fundamentally the structural tensions inherent in organizations that combine regulatory, investigative, and adjudicative functions. When bodies responsible for imposing and enforcing sanctions are also positioned as providers of care, concerns relating to fairness, transparency, and psychological safety may arise, with implications for how legitimacy is perceived by athletes (24, 38). Recent institutional developments, including the delegation of testing and results management functions to bodies such as the International Testing Agency and the Athletics Integrity Unit, have sought to enhance transparency and accountability within the World Anti-Doping Program. However, while these arrangements introduce greater institutional separation from sport governing bodies, they do not resolve the underlying tension between sanctioning authority and care provision, as these organizations remain embedded within the enforcement architecture of the WADC and continue to exercise investigative and regulatory power. Structural independence from sport organizations therefore does not, in itself, address the conflict of interest that arises when sanctioning bodies assume responsibility for athlete support. The findings indicate a need to consider forms of provision that are functionally independent from the sanctioning process altogether, with organizations external to the World Anti-Doping Program potentially better positioned to provide support that is perceived as impartial and safe, while allowing regulatory bodies to retain a clear focus on enforcement and compliance.

Some may argue that post-sanction support undermines deterrence or that committing an ADRV entails forfeiture of assistance. While deliberate doping justifies robust sanctions under strict liability, athletes should not be treated as uniformly malicious, as doping behaviors are shaped by structural pressures, power asymmetries, and normalized risk-taking cultures in elite sport (88–91). These dynamics are particularly evident in systemic doping, where athletes may operate in coercive environments, rendering them simultaneously rule violators and victims [e.g., (92, 93)]. Ethical critiques further note that strict liability separates sanctioning from moral culpability and does not negate welfare obligations (67). Proportionate post-sanction support aligns with broader criminal justice practices, where individuals convicted of serious offences commonly receive structured rehabilitation and reintegration support (94, 95), and may enhance compliance by strengthening procedural legitimacy (2). Yet, no stakeholder is clearly responsible for sanctioned athletes’ welfare. Although the WADC details sanctioning procedures, it does not assign responsibility for psychosocial, medical, or reintegration support (5). This governance gap may compound harms that extend beyond ineligibility to include stigma, identity disruption, and psychological distress, irrespective of intent (96, 97). The gap is especially salient in unintentional ADRVs. Analogous to harm-reduction approaches in recreational drug use policy (83), targeted, Code-compliant support that maintains athletes’ connection to sport may mitigate secondary harms and protect long-term investments in athlete development (98–100).

Addressing this gap would require mapping the transition in duty of care from sanctioning authorities at adjudication through to the completion of a period of ineligibility. Such mapping would clarify which organizations are positioned to provide specific forms of support and enable the development of coordinated, clearly defined pathways. Potential contributors include players associations, which can advocate for athlete interests and provide peer support; national health services and independent mental health providers, which can deliver clinical interventions separate from adjudication processes; clubs and teams, which maintain ongoing relationships with athletes; and independent athlete welfare bodies. Further research should examine the appetite, capacities, and resource limitations of these organizations to determine their readiness and willingness to participate in coordinated care provision. Empirical examination of how athletes experience transitions across different phases of sanctioning would illuminate critical moments when support is most needed. Establishing such frameworks would directly address the welfare gap identified in this review, ensuring that sanctioned athletes receive support that complements the enforcement aims of the WADC and aligns with broader commitments to athlete wellbeing.

### 5.2 Limitations

Several limitations of this review must be acknowledged. As a review of secondary data, the analysis was shaped by what was reported in the included outputs and, therefore, reflects how previous authors have documented athletes’ experiences of the WADC ADRV sanctioning process, rather than providing an assessment of the accuracy of those accounts or offering interpretations beyond what is available in the published evidence base.

The exclusive inclusion of peer-reviewed academic outputs represents a further limitation. While this approach ensured a consistent and standardized level of quality and rigor, it excluded grey literature and other non-peer-reviewed sources, including media accounts, reports produced by advocacy organizations, and legal or disciplinary documents. Perspectives that fall outside academic publishing conventions are therefore underrepresented. Consequently, the findings reflect the perspectives documented within peer-reviewed research and do not encompass the full spectrum of experiences that may exist outside the academic literature.

The restriction to English-language publications constitutes an additional limitation. This decision was determined by the languages spoken within the research team and concerns that selectively including additional languages could introduce bias by privileging particular linguistic contexts. Consequently, the review is likely to have captured fewer culturally and geographically diverse perspectives than have been described within academic literature.

Uncertainty remains regarding whether the same sanctioned athletes contributed to multiple included studies. Recruitment challenges reported across the evidence base indicate that participant overlap cannot be ruled out, meaning that the cumulative findings may reflect the experiences of fewer individuals than aggregate sample sizes suggest.

A further limitation concerns the interpretation of synthesized athlete experiences in relation to anti-doping rule violations. The experiences presented across the included studies should not be read as providing insight into athletes’ lived experiences in relation to intentional or unintentional ADRVs, as this distinction was not consistently reported or analytically preserved at the individual level. Instead, the synthesis reflects broader and often decontextualized accounts of athletes’ perceptions, challenges, and interactions with the WADC sanctioning process and its related impacts, which are not systematically anchored to adjudicated violation outcomes. Three exceptions were identified (24, 32, 42). The first two examined athletes’ lived experiences in relation to ADRVs reported as unintentional, while the latter included athletes who had admitted to intentional violations. Beyond these cases, the remaining studies did not explicitly examine lived experiences in relation to violation intent. Even where aggregate proportions of intentional vs. unintentional ADRVs were reported, this distinction was not preserved at the anonymized individual level. Consequently, specific experiential accounts or verbatim quotations cannot be meaningfully linked to the intentionality of the violation, constraining the interpretive precision of the synthesis. Taken together, the findings are best understood as reflecting general athlete experiences within anti-doping contexts, rather than direct representations of the lived experiences of athletes sanctioned for intentional or unintentional ADRVs.

Finally, the synthesis itself is influenced by factors inherent to the review process. Selection of quotations and thematic coding involve interpretation, and publication bias may affect the available evidence, as peer-reviewed literature could preferentially report cases with more compelling or pronounced findings, potentially underrepresenting less extreme experiences. Variability in how experiences were documented across studies, including differences in qualitative methods, level of detail, and analytic focus, may also influence the comparability and richness of the synthesis.

### 5.3 Recommendations and future research directions

The current review provides a foundational understanding of the lived experiences of athletes exposed to the WADC. However, important opportunities for future research remain. Expanding the range of athlete perspectives following sanctions should be a priority. Limited gender and geographic representation currently constrains insight into how sanctioning processes and their consequences may differ across population groups. Moreover, none of the athletes represented across the twelve outputs were explicitly sanctioned for technical violations such as whereabouts failures or evasion. It therefore remains unclear whether these types of infractions lead to distinct lived experiences. Including a broader range of athletes would allow exploration of underrepresented experiences and provide a stronger foundation for evidence-informed policy development. Expanding the sample beyond previously studied groups would also enable the design of interventions that are better tailored to diverse athlete needs, which is particularly important in a top-down regulatory environment where policies and support mechanisms are standardized. Calls for broader coverage of ADRVs (beyond use and presence), as well as greater geographical representation, have consistently been made in the anti-doping field (51, 88). Capturing a wider array of experiences would allow researchers and policymakers to identify variation in sanction perception, the challenges athletes face, and the supports required, enabling interventions to be more responsive, equitable, and effective across populations.

Enhancing the interpretability of this body of research, while respecting participant anonymity, is a further priority. Academics should carefully balance the reporting of demographic and contextual variables with protection of participant identity. Where ethically feasible, reporting variables such as age at sanction, gender, nationality, geographic location, sport type and level, career stage, procedural details including legal representation during hearings, and whether the athlete returned to sport, could provide valuable context to support evidence synthesis and policy decision-making. Developing guidelines for ethical reporting that balance transparency with protection would support the creation of a more comprehensive, culturally and geographically diverse dataset. Across all research activities, psychological safety, confidentiality, and reciprocal value for participants should be prioritized, with trauma-informed approaches mitigating risks of stigma, reputational harm, or professional repercussions. From a methodological perspective, maintaining rigor when collecting and reporting the perspectives of athletes who have experienced WADC sanctions should incorporate qualitative expertise and adherence to established quality criteria, including credibility, dependability, confirmability, and transferability (101), with the adoption of structured assessment tools potentially assisting with this (31).

Athletes exposed to the WADC remain a hard-to-reach population, as reflected in most outputs included in this review. This underscores the need for methodological innovation beyond traditional social science approaches. Alternative, publicly available sources offer promising avenues for addressing recruitment challenges, including case studies (37, 102), online forums (103, 104), social media (105, 106), print media (107–109), and document analysis (110, 111). Building on these approaches, there may also be scope to introduce formal mechanisms within anti-doping processes that enable athletes to document or communicate experiences in real time. Examples could include anonymized reflective journals, structured debriefs, or confidential testimony submitted to an independent body, which could provide valuable qualitative insight for researchers and policymakers while supporting athletes’ psychological wellbeing.

Future research should also examine organizational and systemic perspectives to build a holistic understanding of the sanctioning ecosystem. Investigating how anti-doping bodies implement policies, provide support, and use research evidence to inform practices may clarify facilitators and barriers to effective duty of care models. Organizational representatives are rarely included in anti-doping research, which limits insight into these processes (112). Understanding whether organizations have drawn on research findings when formulating policies, targeted education programs, or support mechanisms is particularly important. Equally, exploring barriers to consulting existing evidence could provide valuable insight. While the WADC mandates minimum compliance requirements, some organizations have implemented additional measures, for example separately regulating and testing for social drugs (76, 115), providing financial legal aid in some countries (114), offering mental health support for sanctioned athletes, involving former sanctioned athletes as educators and clean sport ambassadors, or delivering additional education for emerging athletes (76, 115). Understanding the extent to which these initiatives are research-based or research-informed, and how they support athletes who access these services, would inform future research and facilitate collaboration between academic researchers and practitioners.

Critically, future research should aim to clarify the purpose and intended function of WADA sanctions, including how sanctions are designed to achieve compliance, deter violations, and uphold legitimacy. Once this understanding has been developed, questions of responsibility for responding to these insights emerge. Whether this responsibility lies with WADA, national agencies, international federations, or a coordinated global approach remains open, but addressing it is necessary for reforms to move beyond acknowledgement toward meaningful action. Integrating organizational insights with a deeper understanding of sanctioning objectives would help ensure that policy and practice reforms are effective, coherent, and aligned with the principles of fairness, deterrence, and legitimacy, while also facilitating more complete policy analysis, which typically involves defining the problem the policy is intended to address and the criteria for evaluating its success (116).

To strengthen the evidentiary foundation of research involving sanctioned athletes, future studies should adopt more transparent and systematic reporting practices within ethical confidentiality. The current lack of detail regarding the nature of ADRVs significantly limits the potential for meaningful comparisons, systematic reviews, and secondary data analysis. Researchers are strongly encouraged to include anonymized participant profile tables that provide essential contextual information. At a minimum, these should include gender, sport, country of residence or nationality, ADRV type, and length of sanction. Additional information, such as career progression after serving an ineligibility period or information on the testing and sanctioning authority, would add further context. Such additions would not only enhance the interpretability of findings but also support the cumulative development of knowledge and evidence-informed policy in the anti-doping field.

Finally, the isolated nature of research on WADC sanctions limits opportunities for theoretical development, longitudinal tracking, and comparative studies (56), leaving the field vulnerable to knowledge silos and slow conceptual progress. Future research could therefore adopt meta-research approaches to examine the structure and processes of the research community itself. Investigating how research is organised, how questions are prioritised, and the barriers to collaboration and knowledge integration could clarify why these structural limitations exist and help identify strategies to foster more coordinated, cumulative, and policy-relevant knowledge production.

## 6 Conclusion

This review synthesized evidence on the experiences of athletes sanctioned under the WADC and their perceptions of the fairness of the sanctioning process. Across 12 outputs, athletes reported diverse physical, psychological, and social consequences, reflecting both immediate and longer-term impacts of ADRV sanctions. The literature is constrained by small samples and recruitment challenges, leaving many athlete experiences underrepresented and limiting understanding of the full scope of sanction-related consequences at both micro (individual) and macro (policy) levels. These findings are critical for WADA and other anti-doping organizations, highlighting how gaps in evidence and understanding can affect the perceived legitimacy of the sanctioning system and its capacity to balance fairness, deterrence, and athlete welfare. As the newly adopted World Anti-Doping Code (coming into effect in 2027) is implemented, it will be essential to evaluate its impact through a human rights lens. Sanctioned athletes, particularly those affected by unintentional rule violations, must be included in such assessments to ensure the system remains not only effective but also equitable and respectful of athletes’ dignity, legal protections, and well-being. Without meaningful inclusion of these voices, the legitimacy and ethical sustainability of anti-doping governance will remain in question. A call to action is clear: future anti-doping policies must be shaped not only by principles of deterrence, but by the lived realities of those most affected.

## Data Availability

The original contributions presented in the study are included in the article/Supplementary Material, further inquiries can be directed to the corresponding author.
