# Supplementary material for: From violation to stigma: a literature review of athletes' lived experiences following anti-doping sanctions
Source: Front Sports Act Living. 2026 Feb 26;8:1651135. doi: 10.3389/fspor.2026.1651135 (PMC12980880; doi:10.3389/fspor.2026.1651135)
Supplement: Supplementary file 1 [file Supplementaryfile1.docx]

Supplementary Material

**Lockett, I., Blank, C., Patterson, L., Westmattelmann, D., Lux, D., Petróczi, A.** From Violation to Stigma: A Literature Review of Athletes’ Lived Experiences Following Anti-Doping Sanctions

# Database Search

# Search Term used for database search: “sport OR athlete OR player; AND “anti-doping rule violation” OR ADRV OR doping OR “whereabout failure” OR “prohibited association” OR “unintentional doping” OR “inadvertent doping” OR possession; AND sanction OR ban OR ineligibility”

# Supplementary Table 1.1: Results of keyword-based database searches

| **Database** | **Total number of records** | **Number of records after filtering for English** | **Number of records after filtering for English and publication years 2003–2025** | **Number of records after filtering for English, 2003–2025, and research articles** | **Number of records after filtering for English, 2003–2025, research articles, and relevant social science topics** |
| --- | --- | --- | --- | --- | --- |
| Web of Science | 1,367,395 | 1,325,401 | 1,146,936 | 898,550 | 166,339 |
| SportsDiscuss | 4 | 3 | 3 | 1 | 1 |
| Scopus | 299 | 260 | 243 | 92 | 92 |
| Dimensions.AI | 435,471 | 435,471  (Filtering by language is not an option) | 378,703 | 84,102 | 31,746 |
| Pubmed | 54,980 | 39,758 | 35,494 | 3,653 | 3,653 (Filtering by relevant social science topics is not an option) |
| Google Scholar (Point of Reference Search) | 4,740 | 4,740  (Filtering by language is not an option) | 4,490 | 4,490  (Filtering by article type is not an option) | 4,490 (Filtering by relevant social science topics is not an option) |

# Quality Assessment

## Supplementary Table 2.1: Detailed scores of all raters including the reference point scores of Rater 6

|  | Rater | Sum | 1. Is there congruity between the stated philosophical perspective and the research methodology? | 2. Is there congruity between the research methodology and the research question or objectives? | 3. Is there congruity between the research methodology and the methods used to collect data? | 4. Is there congruity between the research methodology and the representation and analysis of data? | 5. Is there congruity between the research methodology and the interpretation of results? | 6. Is there a statement locating the researcher culturally or theoretically? | 7. Is the influence of the researcher on the research, and vice- versa, addressed? | 8. Are participants, and their voices, adequately represented? | 9. Is the research ethical according to current criteria or, for recent studies, and is there evidence of ethical approval by an appropriate body? | 10. Do the conclusions drawn in the research report flow from the analysis, or interpretation, of the data? |
| --- | --- | --- | --- | --- | --- | --- | --- | --- | --- | --- | --- | --- |
| Engelberg et al. (2015) | 1 | 6.5 | 0 | 1 | 1 | 1 | 1 | 0 | 0 | 1 | 1 | 0.5 |
|  | 2 | 4 | 0 | 1 | 1 | 0.5 | 0 | 0 | 0 | 0.5 | 1 | 0 |
|  | 3 | 6.5 | 0 | 1 | 1 | 1 | 1 | 0 | 0 | 1 | 1 | 0.5 |
|  | 4 | 6 | 0 | 1 | 1 | 1 | 1 | 0 | 0 | 1 | 1 | 0 |
|  | 5 | 5.5 | 0 | 1 | 1 | 1 | 1 | 0 | 0 | 1 | 1 | 0.5 |
|  |  | 28.5^+^ |  |  |  |  |  |  |  |  |  |  |
| Erickson (2019) | 1 | 9.5 | 1 | 1 | 1 | 1 | 1 | 1 | 0.5 | 1 | 1 | 1 |
|  | 2 | 8 | 1 | 1 | 1 | 1 | 1 | 1 | 0.5 | 1 | 0.5 | 0 |
|  | 3 | 9.5 | 1 | 1 | 1 | 1 | 1 | 1 | 0.5 | 1 | 1 | 1 |
|  | 4 | 10 | 1 | 1 | 1 | 1 | 1 | 1 | 1 | 1 | 1 | 1 |
|  | 5 | 10 | 1 | 1 | 1 | 1 | 1 | 1 | 1 | 1 | 1 | 1 |
|  |  | 47^+^ |  |  |  |  |  |  |  |  |  |  |
| Georgiadis & Papazoglou (2014) | 1 | 7.5 | 1 | 1 | 1 | 1 | 1 | 0 | 0 | 1 | 0.5 | 1 |
|  | 2 | 6 | 1 | 1 | 1 | 0.5 | 0.5 | 0 | 0 | 1 | 0.5 | 0.5 |
|  | 3 | 7.5 | 1 | 1 | 1 | 1 | 1 | 0 | 0 | 1 | 0.5 | 1 |
|  | 4 | 6.5 | 0.5 | 1 | 1 | 1 | 1 | 0 | 0 | 1 | 0 | 1 |
|  | 5 | 7.5 | 1 | 1 | 1 | 1 | 1 | 0 | 0.5 | 1 | 0.5 | 0.5 |
|  |  | 35^+^ |  |  |  |  |  |  |  |  |  |  |
| Hall et al. (2025) | 1 | 10 | 1 | 1 | 1 | 1 | 1 | 1 | 1 | 1 | 1 | 1 |
|  | 2 | 10 | 1 | 1 | 1 | 1 | 1 | 1 | 1 | 1 | 1 | 1 |
|  | 3 | 10 | 1 | 1 | 1 | 1 | 1 | 1 | 1 | 1 | 1 | 1 |
|  | 4 | 10 | 1 | 1 | 1 | 1 | 1 | 1 | 1 | 1 | 1 | 1 |
|  | 5 | 10 | 1 | 1 | 1 | 1 | 1 | 1 | 1 | 1 | 1 | 1 |
|  |  | 50^+^ |  |  |  |  |  |  |  |  |  |  |
| Henning & Dimeo (2014) | 1 | 5 | 0 | 1 | 1 | 1 | 1 | 0 | 0 | 0 |  | 1 |
|  | 2 | 4.5 | 0 | 0.5 | 1 | 1 | 1 | 0 | 0 | 0.5 |  | 0.5 |
|  | 3 | 5 | 0 | 1 | 1 | 1 | 1 | 0 | 0 | 0 |  | 1 |
|  | 4 | 2.5 | 0 | 0 | 1 | 1 | 0.5 | 0 | 0 | 0 |  | 0 |
|  | 5 | 6 | 0 | 1 | 1 | 1 | 1 | 0 | 0.5 | 0.5 |  | 1 |
|  | 6* | 5.5 | 0.5 | 1 | 1 | 1 | 1 | 0 | 0 |  |  | 1 |
|  |  | 23^+^ |  |  |  |  |  |  |  |  |  |  |
| Huseynli et al. (2025) | 1 | 6.5 | 0.5 | 1 | 1 | 1 | 0 | 0 | 0 | 1 | 1 | 1 |
|  | 2 | 5.5 | 0 | 1 | 1 | 1 | 0 | 0 | 0 | 1 | 1 | 0.5 |
|  | 3 | 6.5 | 0.5 | 1 | 1 | 1 | 0 | 0 | 0 | 1 | 1 | 1 |
|  | 4 | 5.5 | 0 | 1 | 1 | 1 | 0 | 0 | 0 | 0.5 | 1 | 1 |
|  | 5 | 6.5 | 0.5 | 1 | 1 | 1 | 0 | 0 | 0 | 1 | 1 | 1 |
|  | 6* | 5.5 | 0.5 | 1 | 1 | 1 | 0 | 0 | 0 | 0 | 1 | 1 |
|  |  | 30.5^+^ |  |  |  |  |  |  |  |  |  |  |
| Juma & Woolf (2026) | 1 | 9.5 | 1 | 1 | 1 | 1 | 1 | 0.5 | 1 | 1 | 1 | 1 |
|  | 2 | 9 | 1 | 0.5 | 1 | 1 | 1 | 0.5 | 1 | 1 | 1 | 1 |
|  | 3 | 10 | 1 | 1 | 1 | 1 | 1 | 1 | 1 | 1 | 1 | 1 |
|  | 4 | 9.5 | 1 | 0.5 | 1 | 1 | 1 | 1 | 1 | 1 | 1 | 1 |
|  | 5 | 9.5 | 1 | 1 | 1 | 0.5 | 1 | 1 | 1 | 1 | 1 | 1 |
|  |  | 47.5^+^ |  |  |  |  |  |  |  |  |  |  |
| Kirby et al. (2011) | 1 | 7.5 | 0.5 | 1 | 1 | 1 | 1 | 0 | 0 | 1 | 1 | 1 |
|  | 2 | 5.5 | 0 | 1 | 1 | 1 | 1 | 0 | 0 | 0.5 | 1 | 0 |
|  | 3 | 7.5 | 0.5 | 1 | 1 | 1 | 1 | 0 | 0 | 1 | 1 | 1 |
|  | 4 | 6 | 0 | 1 | 1 | 1 | 1 | 0 | 0 | 1 | 1 | 0 |
|  | 5 | 6 | 0 | 1 | 1 | 1 | 1 | 0 | 0 | 1 | 1 | 1 |
|  |  | 32.5^+^ |  |  |  |  |  |  |  |  |  |  |
| Piffaretti (2011) | 1 | 5 | 0 | 0.5 | 0.5 | 0.5 | 1 | 0 | 0 | 1 | 0.5 | 1 |
|  | 2 | 3.5 | 0 | 0.5 | 0.5 | 0.5 | 0.5 | 0 | 0 | 0.5 | 0.5 | 0.5 |
|  | 3 | 5.5 | 0 | 0.5 | 0.5 | 0.5 | 1 | 0 | 0 | 1 | 1 | 1 |
|  | 4 | 5 | 0 | 0.5 | 0.5 | 0.5 | 1 | 0 | 0 | 1 | 0.5 | 1 |
|  | 5 | 5 | 0 | 0.5 | 0.5 | 0.5 | 1 | 0 | 0 | 1 | 0.5 | 1 |
|  | 6* | 4 | 0.5 | 0.5 | 0.5 | 0.5 | 1 | 0 | 0 |  | 0 | 1 |
|  |  | 24^+^ |  |  |  |  |  |  |  |  |  |  |
| Shelley (2022) | 1 | 10 | 1 | 1 | 1 | 1 | 1 | 1 | 1 | 1 | 1 | 1 |
|  | 2 | 10 | 1 | 1 | 1 | 1 | 1 | 1 | 1 | 1 | 1 | 1 |
|  | 3 | 10 | 1 | 1 | 1 | 1 | 1 | 1 | 1 | 1 | 1 | 1 |
|  | 4 | 10 | 1 | 1 | 1 | 1 | 1 | 1 | 1 | 1 | 1 | 1 |
|  | 5 | 10 | 1 | 1 | 1 | 1 | 1 | 1 | 1 | 1 | 1 | 1 |
|  |  | 50^+^ |  |  |  |  |  |  |  |  |  |  |
| van der Kallen et al. (2023) | 1 | 5.5 | 0.5 | 1 | 1 | 0 | 0 | 0 | 0 | 1 | 1 | 1 |
|  | 2 | 3.5 | 0.5 | 0.5 | 0.5 | 0 | 0 | 0 | 0 | 1 | 1 | 0 |
|  | 3 | 6 | 1 | 1 | 1 | 0 | 0 | 0 | 0 | 1 | 1 | 1 |
|  | 4 | 4.5 | 0.5 | 1 | 1 | 0 | 0 | 0 | 0 | 0 | 1 | 1 |
|  | 5 | 6.5 | 0.5 | 1 | 1 | 0.5 | 0.5 | 0 | 0 | 1 | 1 | 1 |
|  | 6* | 5 | 1 | 0 | 1 | 0 | 0 | 0 | 0 | 1 | 1 | 1 |
|  |  | 26^+^ |  |  |  |  |  |  |  |  |  |  |

* Reference Score of Rater 6; ^+^  Sum of all raters except reference point rating; 0=No, 1= Yes, 0.5=Unclear, Missing=Not Available; green: total agreement; orange: slight disagreement (“yes” versus “unclear” and “no” versus “unclear”); red: disagreement (at least one “no” versus “yes”)

Two studies (Hall et al., 2025; Shelley et al., 2022) in the sample demonstrated excellent quality (50/50 points). Juma (2024) also demonstrated very high quality with only some of the points unclear (47/50). On the lower end (still >20 out of 50) were Engelberg et al. (2015), Henning & Dimeo (2014), Piffaretti (2011) and Van der Kallen et al. (2023). These studies mostly lacked alignment between the philosophical underpinning and the research methodology, the positioning of the researchers in terms of their cultural and theoretical background, and how these might influence the research. Van der Kallen et al. (2023) additionally showed weaknesses in aligning research methodology, objectives, and data analyses.

The most compelling disagreements between the raters in the first round were with respect to giving “unclear” rating versus a categorical “no”. During discussion it transpired that in the absence of the relevant information, some assessors were willing to fill the gap and assume, whereas others took a stricter position and marked the study down for the missing information. However, most of these conflicts were resolved to a consensus on “no” using the reference point rating and the discussion between the raters.

Disagreement was also related to the question regarding an adequate and data-relevant conclusion. This was mainly because of the different expectations of the authors in terms of what a study conclusion should look like. As such, this section still is the one with a lower agreement between the raters.

## Supplementary Table 2.2: Weighted Cohen-Kappa indicating before discussion and without reference point rater (Rater 6)

| **Ratings** | **Weighted Kappa^a^** | **Asymptotic** | | | **95% asymptotic confidence interval** | |
| --- | --- | --- | --- | --- | --- | --- |
|  |  | **Standard error^b^** | **z^c^** | **Sig.** | **Min** | **Max^b^** |
| Rater_1 - Rater_2 | .511 | .066 | 7.067 | <.001 | .382 | .641 |
| Rater_1 - Rater_3 | .853 | .054 | 9.808 | <.001 | .747 | .958 |
| Rater_1 - Rater_4 | .663 | .085 | 7.414 | <.001 | .496 | .829 |
| Rater_1 - Rater_5 | .627 | .092 | 7.416 | <.001 | .446 | .808 |
| Rater_2 - Rater_3 | .453 | .066 | 6.780 | <.001 | .324 | .582 |
| Rater_2 - Rater_4 | .435 | .079 | 5.515 | <.001 | .281 | .589 |
| Rater_2 - Rater_5 | .312 | .073 | 5.048 | <.001 | .169 | .454 |
| Rater_3 - Rater_4 | .605 | .091 | 6.930 | <.001 | .427 | .783 |
| Rater_3 - Rater_5 | .590 | .103 | 7.006 | <.001 | .388 | .791 |
| Rater_4 - Rater_5 | .476 | .098 | 6.040 | <.001 | .284 | .667 |
| a. Linear weightings are used to estimate the weighted kappa. | | | | | | |
| b. The value depends neither on null hypotheses nor on alternative hypotheses. | | | | | | |
| c. Estimates of the asymptotic standard error assuming the null hypothesis that the weighted kappa is zero. | | | | | | |

## Supplementary Table 2.3: Weighted Cohen-Kappa indicating after discussion and with reference point rater (Rater 6)

| **Ratings** | **Weighted Kappa^a^** | **Asymptotic** | | | **95% asymptotic confidence interval** | |
| --- | --- | --- | --- | --- | --- | --- |
|  |  | **Standard error^b^** | **z^c^** | **Sig.** | **Min** | **Max^b^** |
| Rater_1 - Rater_2 | .693 | .060 | 8.801 | <.001 | .575 | .811 |
| Rater_1 - Rater_3 | .961 | .022 | 11.164 | <.001 | .918 | 1.005 |
| Rater_1 - Rater_4 | .792 | .055 | 9.294 | <.001 | .684 | .900 |
| Rater_1 - Rater_5 | .865 | .040 | 10.377 | <.001 | .788 | .943 |
| Rater_1 - Rater_6 | .780 | .088 | 5.520 | <.001 | .608 | .952 |
| Rater_2 - Rater_3 | .660 | .062 | 8.412 | <.001 | .538 | .781 |
| Rater_2 - Rater_4 | .686 | .060 | 8.654 | <.001 | .568 | .804 |
| Rater_2 - Rater_5 | .633 | .064 | 8.293 | <.001 | .509 | .758 |
| Rater_2 - Rater_6 | .538 | .106 | 4.137 | <.001 | .329 | .747 |
| Rater_3 - Rater_4 | .778 | .056 | 9.069 | <.001 | .668 | .888 |
| Rater_3 - Rater_5 | .849 | .042 | 10.095 | <.001 | .767 | .932 |
| Rater_3 - Rater_6 | .781 | .094 | 5.454 | <.001 | .596 | .966 |
| Rater_4 - Rater_5 | .733 | .058 | 8.921 | <.001 | .619 | .848 |
| Rater_4 - Rater_6 | .597 | .110 | 4.330 | <.001 | .381 | .813 |
| Rater_5 - Rater_6 | .691 | .094 | 5.102 | <.001 | .508 | .875 |
| a. Linear weightings are used to estimate the weighted kappa. | | | | | | |
| b. The value depends neither on null hypotheses nor on alternative hypotheses. | | | | | | |
| c. Estimates of the asymptotic standard error assuming the null hypothesis that the weighted kappa is zero. | | | | | | |

1. **Keywords, Key Terms and Expertise**

**Supplementary Table 3.1: Author’ Keywords, AI-generated Key Terms and Authors’ Expertise**

| **Reference** | **Source** | **Author’s keywords** | **Dimensions.AI top keywords** | **Research team expertise** |
| --- | --- | --- | --- | --- |
| Engelberg, T., Moston, S., & Skinner, J. (2015) | Journal article | Doping; Drugs in Sport; Moral Disengagement; Deterrence; Policy | Doping; Athletes; Moral disengagement; Normalization; Anti-doping policy; Deterrents; Experiences; Psychology; Violations; Sports | Sport management  Psychology  Sports Business |
| Erickson, K. (2019) | Journal article | Doping; Student-Athlete; Risk  Factors; Narrative; Sanctioned  Athlete | Banned substance;  Student-athlete; Doping sanction; Risk factors; Narrative approach; Supplementation;  Injury; Family life;  Protection | Sport psychology |
| Georgiadis, E. & Papazoglou, I. (2014) | Journal article | Not available | Competition ban;  Doping;  Elite athletes;  Psychological implications; Social implications;  Financial implications;  World Anti-Doping Agency (WADA);  Doping violation;  Mental health;  Qualitative analysis; | Sport and Exercise  Psychology  Clinical Psychology |
| Hall, J., Patterson, L., & Backhouse, S. (2025) | Journal article | Doping violations; Substance abuse; Rugby;  Social identity theory; Creative non-fiction | Doping; Rugby; Anti-doping rules; Sanctioned athletes; Composite vignettes; Lived experiences; Social identity; Prevention; Rehabilitation; Critical incidents | Sport and Exercise Psychology |
| Henning, A. & Dimeo, P. (2014) | Journal article | Amateur; Anti-Doping; Cycling; Doping; Sport; United States | Anti-doping Violations; USA cycling; Banned substances; Sanctions; WADA (World Anti-Doping Agency); Athlete health; Typology of cases; Amateur athletes; Masters athletes | Sociology  Sport history |
| Huseynli, S., Lazuras, L., Petrou, M., Abasov, F., & Bingham, K. (2025) | Journal article | Anti-Doping Proceedings; Procedural Fairness; Legitimacy; Human Rights; Athletes | Procedural fairness; Perceived legitimacy; Anti-doping proceeding; National Anti-Doping Organizations; Due process; Sanctioned athletes; Athlete representatives; Mixed methods; Legal awareness; Human rights protection | Policy development  Sport and exercise science  Medicine  Law  Psychology |
| Jamieson, V., & Ordway, C, (2021) | Book Chapter | N/A | Not available | Education  Law and Ethics |
| Juma, B., & Woolf, J. (2026) | Journal article | Family dynamics; Mental health; Anti-doping sanctions; Stigma; Suicidality | Doping sanctions; Kenyan athletes; Psychological distress; Social isolation; Financial hardship; Anti-doping rules; Personal effects; Professional impacts; Sport integrity; Reintegration | Sport ethics  Sport Sciences |
| Kirby, K., Moran, A., & Guerin, S. (2011) | Journal article | Performance-Enhancing Drugs; Doping in Sport; Drugs; Psychology; Elite Athletes; Anti-Doping Policy | Doping; Elite athletes; Performance enhancement; Psychological factors Contextual influences; Thematic analysis; Morality; Anti-doping policy; Personal consequences; Social consequences | Psychology  Psychology  Applied health research and evaluation |
| Piffaretti, M. (2011) | Research Grant Report | N/A | Not available | Sport Psychology |
| Shelley, J. (2022) | PhD Thesis chapter | N/A | Not available | Biochemistry & Kinesiology |
| van der Kallen, Lux, Schobersberger, Kleiner, Eisenburger, Blank | Journal article | Interview Guide; Doping Offence; Athlete’s Health; Support Programs | Doping; Anti-doping rule violation;  Athletes' health;  Biopsychosocial changes;  Psychological well-being;  Interview guide;  Sanctions;  Professional situation;  Social environment;  Physical condition | Psychiatry and Psychotherapy  Sports Science  Sports medicine  Exercise and Sport  Public Health |

1. **Citation analysis**

**Supplementary Table 4.1: Citation Trends over Time**

|  | **2011** | **2012** | **2013** | **2014** | **2015** | **2016** | **2017** | **2018** | **2019** | **2020** | **2021** | **2022** | **2023** | **2024** | **2025** |
| --- | --- | --- | --- | --- | --- | --- | --- | --- | --- | --- | --- | --- | --- | --- | --- |
| Engelberg et al., 2014 |  |  |  | 3 | 1 | 10 | 8 | 6 | 10 | 12 | 11 | 11 | 5 | 9 | 2 |
| Erickson, 2019 |  |  |  |  |  |  |  |  | 0 | 0 | 0 | 0 | 0 | 0 | 1 |
| Georgiadis & Papazoglou, 2014 |  |  |  | 0 | 0 | 3 | 2 | 1 | 2 | 1 | 2 | 1 | 3 | 2 | 2 |
| Hall et al., 2025 |  |  |  |  |  |  |  |  |  |  |  |  |  |  | 0 |
| Henning & Dimeo, 2014 |  |  |  | 0 | 0 | 4 | 1 | 2 | 0 | 2 | 3 | 0 | 0 | 1 | 0 |
| Huseynli et al., 2025 |  |  |  |  |  |  |  |  |  |  |  |  |  |  | 0 |
| Juma & Woolf, 2026 |  |  |  |  |  |  |  |  |  |  |  |  |  |  | 0 |
| Kirby et al. 2011 | 1 | 2 | 6 | 11 | 7 | 13 | 7 | 10 | 11 | 7 | 8 | 12 | 11 | 4 | 2 |
| van der Kallen et al., 2023 |  |  |  |  |  |  |  |  |  |  |  |  | 0 | 0 | 2 |

**Supplementary Table 4.2: Quantitative indicators of academic impact** (Source: Dimensions.AI: Clarivate (WoS), and Scopus; note: citations by year are provided in Supplementary Table 4.1)

|  | **Number of citations (Dimensions.AI)** | **Dimensions.AI field citation ration (expected: 1.0)** | **Number of citations (Scopus)** | **Field-Weighted citation impact (Scopus)** | **Number of citations (Clarivate)** | **Category citation impact (Clarivate)** | **Altmetric score** |
| --- | --- | --- | --- | --- | --- | --- | --- |
| Engelberg et al., 2014 | 88 | 23.00 | 86 (95^th^ percentile) | 3.93 | 79 | Above average | 13 |
| Erickson, 2019 | 1 | 0.33 | Not found | Not found | 1 | Below average | 5 |
| Georgiadis & Papazoglou, 2014 | 19 | 3.16 | 16 (55^th^ percentile) | 0.5 | Not found | Not found | 0 |
| Hall et al., 2025 | 0 | Not yet available | Not yet available | Not yet available | Not yet available | Not yet available | 1 |
| Henning & Dimeo, 2014 | 13 | 2.56 | 13 (60^th^ percentile) | 0.63 | Not found | Not found | 14 |
| Huseynli et al., 2025 | 0 | Not yet available | Not yet available | Not yet available | Not yet available | Not yet available | 1 |
| Juma & Woolf, 2026 | 0 | Not yet available | Note yet available | Not yet available | Not yet available | Not yet available | 10 |
| Kirby et al. 2011 | 112 | 23.00 | 108 (94^th^ percentile) | 3.69 | Not found | Not found | 5 |
| van der Kallen et al., 2023 | 2 | 1.76 | 2 (58^th^ percentile) | 0.59 | Not found | Not found | 5 |

**Supplementary Table 4.3: Impact by field** (Source: Dimensions.AI^a^: Clarivate (WoS), and Scopus)

|  | **Dimensions Citation by field^b^** | **Scopus citation by field** | **Clarivate (WoS) citation by field^c^** | **PlumX metrics (Scopus)** | **Altmetric scores by source** |
| --- | --- | --- | --- | --- | --- |
| Engelberg et al., 2014 | Health Sciences (30.1%)  Commerce, Management, Tourism and Services (18.7%)  Psychology (16.3%)  Human Society (14.6%)  Biomedical and Clinical Sciences (7.3%)  Language, Communication and Culture (4.9%)  Philosophy and Religious Studies (2.4%)  Information and Computing Sciences (1.6%)  Chemical Sciences (0.8%)  Creative Arts and Writing (0.8%)  Education (0.8%)  Engineering (0.8%)  Law and Legal Studies (0.8%) | Social Sciences (25.7%)  Medicine (%)  Health Professionals (15.0%)  Business, Management, and Accounting (12.0%)  Psychology (12.0%)  Decision Science (5.4%)  Biochemistry, Genetics, and Molecular Biology (3.6%)  Economics, Econometrics, and Finance (1.8%)  Chemistry (1.2%)  Environmental Science (1.2%)  Other (2.4%) | Sport Sciences (68.5%)  Social Sciences Other Topics (61.6%)  Psychology (57.5%)  Behavioural Sciences (34.3%)  Toxicology (26.0%)  Sociology (23.3%)  Business Economics (21.9%)  Health Care Sciences Services (17.8%)  Substance Abuse (17.8%)  Paediatrics (6.9%)  Public Administration (6.9%)  Education Educational Research (5.5%)  Government Law (5.5%)  Pathology (5.5%)  Pharmacology Pharmacy (5.5%)  Communication (4.1%)  Environmental Sciences Ecology (4.1%)  General Internal Medicine (4.1%)  Chemistry (2.7%)  Computer Science (2.7%)  Criminology Penology (2.7%)  Geography (2.7%)  Psychiatry (2.7%)  Science Technology Other Topics (2.7%)  Anthropology (1.4%)  Arts Humanities Other Topics (1.4%)  Biochemistry Molecular Biology (1.4%)  Cultural Studies (1.4%)  Demography (1.4%)  Film Radio Television (1.4%)  History (1.4%)  Medical Ethics (1.4%)  Operations Research Management Science (1.4%)  Physiology (1.4%)  Public Environmental Occupational Health (1.4%)  Rehabilitation (1.4%) | 49 captures (readers)  44 Citation Indexes  3 Policy Citations (in 1 document: Study on the fight against anabolic steroids and human growth hormones in sport within the EU: a report to the European Commission, 2021) | High Attention Score  In the top 25% of all research outputs scored by Altmetric  Mentioned by 1 policy source  And 12 twitter users  88 Dimensions citation  150 readers on Mendeley |
| Erickson, 2019 | Health Sciences (100%) | Not found | Not available | Not found | Good Attention  Score compared to outputs of the same age (67th percentile)  Mentioned by 8 twitter users  1 Dimensions citation  12 Readers on Mendeley |
| Georgiadis & Papazoglou, 2014 | Health Sciences (22.7%)  Commerce, Management, Tourism and Services (22.7%)  Psychology (22.7%)  Human Society (13.64%)  Biomedical and Clinical Sciences (9.01%)  Information and Computing Sciences (4.6%)  (Language, Communication and Culture (4.6%) | Social sciences (24.1%)  Medicine (20.7%)  Psychology (20.7%)  Health professional (13.8%)  Business, Management and Accounting (10.3%)  Biochemistry (3.4%)  Decision Science (3.4%)  Multidisciplinary (3.4%) | Not found | 32 captures (readers)  8 citation indexes | None |
| Hall et al., 2025 | Not yet available | Not yet available | Not yet available | 3 captures (readers) | Not yet available |
| Henning & Dimeo, 2014 | Health Sciences (33.3%)  Human Society (33.34&)  Biomedical and Clinical Sciences (13.3%)  Commerce, Management, Tourism and Services (13.3%)  Psychology (6.7%) | Medicine (33.3%)  Social Sciences (33.3%)  Health Professions (12.5%)  Biochemistry, Genetics, and Molecular Biology (4.2%)  Business, Management, and Accounting (4.2%)  Chemistry (4.2%)  Pharmacology, Toxicology, and Pharmaceutics (4.2%)  Psychology (4.2%) | Not found | 42 captures (readers)  7 Citation Indexes  24 Shares, Likes & Comments | High Attention Score  In the top 25% of all research outputs scored by Altmetric |
| Huseynli et al., 2025 | Not yet available | Not yet available | Not yet available | Not yet available | Average Attention Score  Mentioned by 1 Bluesky user |
| Juma & Woolf, 2026 | Not yet available | Not yet available | Not yet available | Not yet available | In the top 25% of all research outputs scored by Altmetric  1 News  Mentioned by 1 X/Twitter |
| Kirby et al. 2011 | Health Sciences (27.3%)  Psychology (19.4%)  Commerce, Management, Tourism and Services (17.6%)  Human Society (15.15%)  Biomedical and Clinical Sciences (8.5%)  Law and Legal Studies (3.0%)  Philosophy and Religious Studies (2.4%)  Engineering (1.8%)  Language, Communication and Culture (1.8%)  Economics (1.2%)  Education (1.2%)  Information and Computing Sciences (0.6%) | Social Sciences (26.4%)  Medicine (21.4%)  Psychology (17.4%)  Health Professions (16.9%)  Business, Management, and Accounting (8.0%)  Arts and Humanities (3.0%)  Decision Science (3.0%)  Biochemistry, Genetics, and Molecular Biology (2.0%)  Economics, Econometrics, and Finance (1.0%)  Multidisciplinary (0.5%)  Other (0.5%) | Not found | 120 Captures (readers)  87 Citation Indexes | Good Attention Score  Mentioned by 6 X/Twitter users,  Dimensions citations,  122 Mendeley readers |
| van der Kallen et al., 2023 | Health Sciences (100%) | Social Science (33.3%)  Medicine (33.3%)  Health professions (33.3%) | Not found | 14 captures (readers)  49 social (shares, likes and comments) | Good Attention Score  Mentioned by 8 X/Twitter users  Citations  2 Dimensions citations  17 Mendeley readers |

^a^ Citation data or Altmetric scores are not available for book chapters, PhD theses or research reports, thus these outputs (Jamieson & Ordway, 2021; Juma, 2024; Piffaretti, 2011; Shelley, 2022) are omitted from this table.

^b^ Dimensions .AI uses the Fields of Research (FoR) codes, which are part of the Australian and New Zealand Standard Research Classification (ANZSRC) system. It is hierarchically structured and designed to categorize research activities based on *methodology* and *focus*

**Supplementary Table 4.4: Citation types**

| **Output (cites)** | **Output (cited)** | **Citation type** | **Citation evaluation** |
| --- | --- | --- | --- |
| Georgiadis & Papazoglou (2014) | Kirby et al. (2011) | Introduction: motivation and factors contributing to doping  Discussion: support (negative health consequences) | Substantive relevant |
|  | Piffaretti (2011) | Discussion: social identity, lack of financial support, impact on career, long lasting negative psychological impact | Substantive relevant |
| Engelberg et al. (2015) | Kirby et al. (2011) | Introduction: motivation and factors contributing to doping; and difficulty in recruitment  Method: Interview guide  Discussion: support (how doping athletes perceive their own behavior, negative health impacts) and contrast (reasons for doping) | Substantive relevant |
| Henning & Dimeo (2016) | Engelberg et al. (2015) | Discussion: contrasting (motivation for doping) | Substantive but not relevant |
| Erickson (2019) | Kirby et al. (2011) | Mention in the introduction | Cursory |
|  | Piffaretti (2011) | Mention in the introduction | Cursory |
|  | Georgiadis & Papazoglou (2014) | Mention in the introduction | Cursory |
|  | Engelberg et al. (2015) | Mention in the introduction | Cursory |
| Jamieson & Ordway (2021) |  | Not cited any of the included outputs | None |
| Shelley (2022) | Kirby et al. (2011) | Mentions (study of sanctioned athletes; study of admitted dopers) | Cursory |
|  | Georgiadis & Papazoglou (2014) | Mentions (study of sanctioned athletes; study of admitted dopers) | Cursory |
|  | Engelberg et al. (2015) | Mentions (study of admitted dopers) | Cursory |
| van der Kallen et al. (2023) | Georgiadis & Papazoglou (2014) | Introduction: review of past relevant research, lack of specific focus on health as limitation was identified, critiqued for the sample being very specific in terms of origin (all Greek), level of competition and the fact that none of the athletes admitted doping  Methods: inform formulating the interview guide  Discussion: comparing findings; confirming both negative and positive experiences; social and financial impact, and stress | Substantive (relevant) |
|  | Kirby et al. (2011) | Introduction: review of past relevant research, lack of specific focus on health as limitation was identified  Methods: inform formulating the interview guide  Discussion: comparing findings; confirming both negative and positive experiences | Substantive (relevant) |
|  | Piffaretti (2011) | Introduction: review of past relevant research, lack of specific focus on health as limitation was identified; critiqued for having only male athletes and small sample size  Methods: inform formulating the interview guide  Discussion: comparing findings, confirming both negative and positive experiences | Substantive (relevant) |
| Juma & Woolf, 2026 | Kirby et al. (2011) | Introduction: Difficulty in recruitment; motivation for and factor contributing to doping  Discussion: Difficulty in recruitment; social impact of sanctions, stigmatization | Substantive (relevant) |
|  | Piffaretti (2011) | Introduction: challenges athletes face after ADRV; rationale for the study  Methods: Discussion: confirming similar experiences, psychological impact, stigma, financial impact; importance of support network as well as positive impact through life outside sport, increased anti-doping knowledge and risk of supplement use; confirming support needs, contrasting (financial impact and income), building on previous findings | Substantive (relevant) |
|  | Georgiadis & Papazoglou (2014) | Introduction: challenges athletes face after ADRV; rationale for the study  Discussion: confirming (similar experiences; stress and depression, health problems); contrasting (financial impact and income); building on previous findings | Substantive (relevant) |
|  | Engelberg et al. (2015) | Introduction, Method and Discussion: Difficulty in recruitment | Substantive (limited relevance) |
|  | van der Kallen et al. (2023) | Method | Substantive (method informing interview guide) |
| Hall et al. (2025) | Kirby et al. (2011) | Introduction: factors contributing to doping; recognition of previous studies, and difficulty in recruitment | Substantive (limited scope) |
|  | Engelberg et al. (2015) | Introduction: factors contributing to doping; recognition of previous studies, and difficulty in recruitment | Substantive (limited scope) |
|  | Erickson (2019) | Introduction: factors contributing to or protective against doping; | Substantive (not relevant) |
| Huseynli et al. (2025) |  | Not cited any of the included outputs | None |
